# Deep learning applications for human embryo assessment using time-lapse imaging: scoping review

**DOI:** 10.3389/frph.2025.1549642

**Published:** 2025-04-08

**Authors:** Rawan AlSaad, Leen Abusarhan, Nour Odeh, Alaa Abd-alrazaq, Fadi Choucair, Rachida Zegour, Arfan Ahmed, Sarah Aziz, Javaid Sheikh

**Affiliations:** ^1^ AI Center for Precision Health, Weill Cornell Medicine-Qatar, Doha, Qatar; ^2^ Faculty of Medicine, Hashemite University, Zarqa, Jordan; ^3^ Reproductive Medicine Unit, Sidra Medicine, Doha, Qatar; ^4^ Faculty of Exact Sciences, University of Bejaia, Bejaia, Algeria

**Keywords:** artificial intelligence, deep learning, embryo quality, embryo selection, *in vitro* fertilization, IVF, reproductive, women's health

## Abstract

**Background:**

The integration of deep learning (DL) and time-lapse imaging technologies offers new possibilities for improving embryo assessment and selection in clinical *in vitro* Fertilization (IVF).

**Objectives:**

This scoping review aims to explore the range of deep learning model applications in the evaluation and selection of embryos monitored through time-lapse imaging systems.

**Methods:**

A total of 6 electronic databases (Scopus, MEDLINE, EMBASE, ACM Digital Library, IEEE Xplore, and Google Scholar) were searched for peer-reviewed literature published before May 2024. We adhered to the PRISMA guidelines for reporting scoping reviews.

**Results:**

Out of the 773 articles reviewed, 77 met the inclusion criteria. Over the past four years, the use of DL in embryo analysis has increased rapidly. The primary applications of DL in the reviewed studies included predicting embryo development and quality (61%, *n* = 47) and forecasting clinical outcomes, such as pregnancy and implantation (35%, *n* = 27). The number of embryos involved in the studies exhibited significant variation, with a mean of 10,485 (SD = 35,593) and a range from 20 to 249,635 embryos. A variety of data types have been used, namely images of blastocyst-stage embryos (47%, *n* = 36), followed by combined images of cleavage and blastocyst stages (23%, *n* = 18). Most of the studies did not provide maternal age details (82%, *n* = 63). Convolutional neural networks (CNNs) were the predominant deep learning architecture used, accounting for 81% (*n* = 62) of the studies. All studies utilized time-lapse video images (100%) as training data, while some also incorporated demographics, clinical and reproductive histories, and IVF cycle parameters. Most studies utilized accuracy as the discriminative measure (58%, *n* = 45).

**Conclusion:**

Our results highlight the diverse applications and potential of deep learning in clinical IVF and suggest directions for future advancements in embryo evaluation and selection techniques.

## 1 Introduction

Infertility affects approximately 17.5% of the global adult population, with roughly one in six individuals experiencing infertility issues during their lifetime (1). *in vitro* fertilization (IVF) is a widely used fertility treatment that involves ovarian stimulation, oocyte retrieval, fertilization, and embryo culture in controlled conditions before either transferring the embryos to the uterus or cryopreserving them for future treatments. Despite advancements in IVF and related technologies, the success rates per cycle remain relatively low, with significant variations depending on patient and treatment characteristics (2, 3).

Recently, many clinics have adopted time-lapse imaging (TLI) systems, an emerging technology that allows for the continuous monitoring and recording of detailed and dynamic information on embryonic development while maintaining stable culture conditions (4, 5). A TLI system includes an incubator with an integrated microscope and cameras connected to an external computer. Embryo images are captured at defined intervals and at various focal planes throughout the culture period. These images are compiled into a video, enabling detailed morphological and morphokinetic evaluations of embryo development.

Embryo assessment is a critical yet challenging step in IVF, as improving the ability to select embryos with the highest implantation potential can increase pregnancy rates (6). However, conventional evaluation methods face several limitations.

Manual grading is subjective and prone to inter-observer variability, leading to inconsistent assessments. Static morphological grading systems, such as Gardner's blastocyst grading, provide only limited predictive insights, as they evaluate embryos at a single time point rather than tracking developmental patterns. Morphokinetic analysis, which monitors cell division timings, adds predictive value but remains labor-intensive, inconsistent, and difficult to standardize across clinics. Furthermore, manual evaluations are not scalable for high-throughput IVF settings, requiring significant time and expertise. These limitations contribute to suboptimal embryo selection and lower IVF success rates (7). Therefore, the availability of more automated, objective, accurate, cost-effective, and user-friendly software for embryo assessment and selection using time-lapse imaging data could significantly empower embryologists to make more efficient clinical decisions.

**Figure 1:**
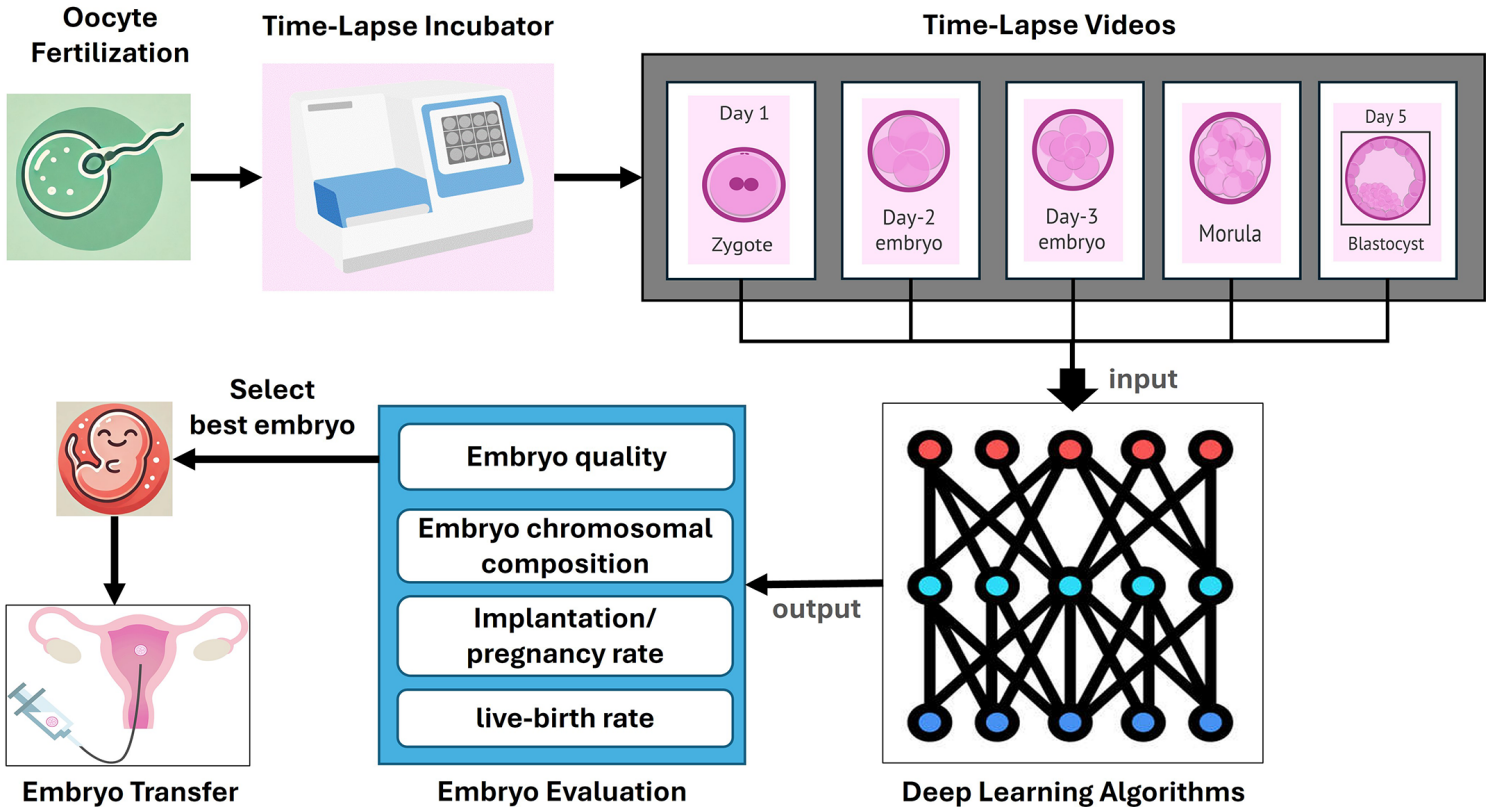
Process of embryo evaluation using time-lapse imaging and deep learning. This figure illustrates the comprehensive process of embryo evaluation in clinical *in vitro* Fertilization (IVF) using time-lapse imaging and deep learning algorithms. The process begins with oocyte fertilization, where the sperm fertilizes the egg, leading to the formation of a zygote. The zygote is then placed in a time-lapse incubator that continuously monitors its development. The timelapse incubator captures sequential images of the embryo at various stages of development, including Day 1, Day 2, Day 3, Day 4, and Day 5 (blastocyst). These images are compiled into time-lapse videos that provide a detailed record of the embryo's development over time. The timelapse videos are then inputted into deep learning algorithms, which analyze the data to evaluate various parameters of the embryos. The deep learning models are trained to assess embryo quality, chromosomal composition, implantation and pregnancy rates, and live-birth rates. The output from these algorithms helps in the selection of the best embryo for transfer. Finally, the selected embryo is transferred to the uterus, where it has the potential to develop into a successful pregnancy.

The emergence of artificial intelligence (AI) technologies and computational methods, such as deep learning (DL), offers promising solutions for overcoming challenges in embryo assessment at different embryonic developmental stages, potentially increasing IVF success rates (8) (Figure 1). Deep neural networks, particularly convolutional neural networks (CNN), provide an efficient alternative to traditional computer vision-based approaches and have shown great promise in biomedical and diagnostic applications (8, 9). This is mainly due to their ability to automate embryo assessment, which eliminates inter- and intra-observer variances and allows for the analysis of vast amounts of data. By identifying subtle patterns that may be overlooked by humans, these DL models can offer more accurate predictions of embryo viability and implantation potential (10–12).

Previous reviews (9, 13–16) on artificial intelligence models in embryology provide valuable insights yet have several significant limitations that this current review aims to address. Firstly, most recent reviews (9, 13–16) were generic, covering a broad range of machine learning models and computer vision techniques, rather than focusing specifically on deep learning algorithms, which are more sophisticated and potentially more effective. Secondly, many of these studies (13, 15) were narrative reviews that did not adhere to a systematic approach, limiting the reproducibility and reliability of their findings. Additionally, previous reviews (9, 13–16) often included a combination of static images and time-lapse video images, whereas our review exclusively focuses on time-lapse images, which can provide more dynamic and comprehensive insights into embryo development. Furthermore, the number of studies included in previous reviews (9, 13–16) was very limited, with a maximum of 30 studies, which could affect the depth and breadth of their conclusions. In contrast, our review specifically addresses the application of deep learning within the context of time-lapse imaging, providing a more focused and up-to-date synthesis of available studies. We expand on previous work by including a significantly larger number of studies (*n* = 77), thereby offering a more comprehensive analysis of recent advancements in deep learning-based embryo evaluation. Additionally, our review systematically examines key characteristics of deep learning models, including their architectures, training methodologies, and validation strategies, providing insights that were previously overlooked in the literature. By refining the scope to exclusively analyze time-lapse imaging applications, we highlight the unique characteristics of AI model architectures and applications associated with using dynamic imaging data for embryo assessment, further reinforcing the relevance of AI-based approaches in reproductive medicine.

In this paper, we aim to provide a focused and comprehensive review of the application of deep learning and time-lapse imaging in embryo assessment. Specifically, this review seeks to answer the following research questions:

1. What are the prevalent applications of deep learning techniques in embryo evaluation and selection using time-lapse imaging?
2. What are the key characteristics of deep learning models used for embryo evaluation and selection, including model architectures, training data types, validation methods, and evaluation metrics?
3. What are the specific characteristics of the embryo populations and time-lapse imaging platforms used for training deep learning models?
4. What future directions can enhance deep learning solutions to meet the needs of IVF timelapse embryology and facilitate the translation of research into clinical practice?
5. By synthesizing these aspects, this review aims to provide a comprehensive understanding of the current state and potential of deep learning applications in embryo evaluation and selection.

## 2 Methods

To achieve the objectives of this study, we carried out a scoping review in accordance with the Preferred Reporting Items for Systematic Reviews and Meta-Analyses - Extension for Scoping Reviews (PRISMA-ScR) guidelines (17). The PRISMA-ScR Checklist associated with this review is available in Supplementary Appendix 1. The subsequent sections provide a comprehensive description of the methods used in this review.

### 2.1 Search strategy

To identify relevant studies, we conducted searches across six electronic databases on November 10, 2023: Scopus, MEDLINE (*via* Ovid), EMBASE (*via* Ovid), ACM Digital Library, IEEE Xplore, and Google Scholar. These databases were selected based on their extensive coverage of medical, computational, and engineering literature, ensuring a comprehensive and multidisciplinary search. Specifically, MEDLINE and EMBASE were chosen for their authoritative indexing of biomedical and clinical research, Scopus for its broad interdisciplinary scope, ACM Digital Library and IEEE Xplore for their focus on AI and computer vision applications, and Google Scholar to capture additional gray literature and emerging studies not indexed in traditional databases. Following this, we set up a biweekly automatic search to run for seven months, ending on May 28, 2024. Due to the high volume of results from Google Scholar, which ranks by relevance, we reviewed only the first 100 entries (10 pages). To ensure a comprehensive review, we also screened the reference lists of our primary selected studies (backward reference checking) and included studies that cited our primary selections (forward reference checking).

Our search query consisted of three primary categories of terms: terms related to Deep Learning (e.g., artificial intelligence, deep learning, convolutional neural network, recurrent neural network), terms related to Time-lapse Imaging (e.g., time-lapse), and terms related to IVF (e.g., *in vitro* fertilization, assisted reproductive technologies, and intracytoplasmic sperm injection).

These categories were structured to ensure the inclusion of all studies that focus on AI applications in embryo assessment using time-lapse imaging while minimizing irrelevant results. Detailed search queries for each database are provided in Supplementary Appendix 2.

### 2.2 Study eligibility criteria

This review focused on studies that investigated the use of deep learning and time-lapse imaging for embryo assessment in the IVF embryology labs. The eligibility criteria were meticulously developed to ensure a comprehensive and focused review of the relevant literature for our study.

#### 2.2.1 Inclusion criteria

Studies were considered eligible if they employed deep learning methods for embryo assessment using time-lapse imaging to monitor embryo development. Additionally, studies needed to report data on the performance (e.g., accuracy) of the applied deep learning methods and involve human embryos undergoing IVF procedures with a focus on embryo evaluation. Studies that also compared deep learning methods to manual preselection by embryologists were included. We included studies with endpoints related to predictions of embryo morphology or outcomes, including embryo stage classification, blastocyst morphology quality and grading, embryo ploidy, and IVF success rates such as implantation rate, clinical pregnancy, and live birth rates. In terms of study design, we included both retrospective and prospective studies. Eligible publications encompassed peer-reviewed articles, theses, dissertations, and conference articles, all of which were required to be published in English. There were no constraints on the year of publication, age groups, or ethnicities.

#### 2.2.2 Exclusion criteria

We excluded studies that involved nonhuman subjects or those that used static images rather than time-lapse imaging for monitoring embryos. Additionally, studies were excluded if they did not deploy deep learning techniques or relied solely on traditional machine learning models (e.g., support vector machines, decision trees, or random forests) or statistical models. Studies lacking sufficient details about the specific role of the deep learning technique in the embryo evaluation process were also excluded. Furthermore, we excluded studies focusing on outcomes related to embryo culture medium analysis, as well as those examining post-IVF cycle outcomes, such as neonatal health and complications. Regarding the type of publication, we excluded non-peerreviewed articles, preprints, reviews, opinion papers, research letters, commentaries, editorials, case studies, conference abstracts, posters, and protocols.

### 2.3 Study selection

The study selection process was conducted in three phases. Initially, duplicates were removed from the retrieved studies using EndNote X9. Subsequently, we screened the titles and abstracts of the remaining articles. In the final phase, the full texts of the shortlisted studies were thoroughly evaluated. The selection process was independently conducted by two reviewers, with any disagreements resolved by consultation with a third reviewer. To evaluate the level of agreement between the two reviewers, we used Cohen's kappa (18). The resulting kappa values were 0.66 for title and abstract screening and 0.78 for full-text screening, indicating substantial agreement.

### 2.4 Data extraction

Two reviewers independently used Microsoft Excel to collect data on study metadata, study design, embryology and time-lapse characteristics, and AI methods. Any disagreements were resolved through discussion. The data extraction form for this review was piloted with ten studies and is available in Supplementary Appendix 3.

### 2.5 Data synthesis

We synthesized the extracted data from the included studies using a narrative approach, providing a comprehensive summary through text, tables, and figures. Initially, we described the characteristics of the included studies, covering aspects such as publication characteristics, study type and sites, participants, and data sources. Next, we detailed the applications and outcomes, including both main and specific applications as well as the outcome measures used in the studies. We then summarized the embryology and time-lapse imaging characteristics, focusing on embryo populations, the time-lapse platform employed, and the annotation standards used. Finally, we detailed the deep learning model characteristics, including the architectures of the deep learning algorithms, input data types, validation methods, and performance metrics.

## 3 Results

### 3.1 Search results

**Figure 2:**
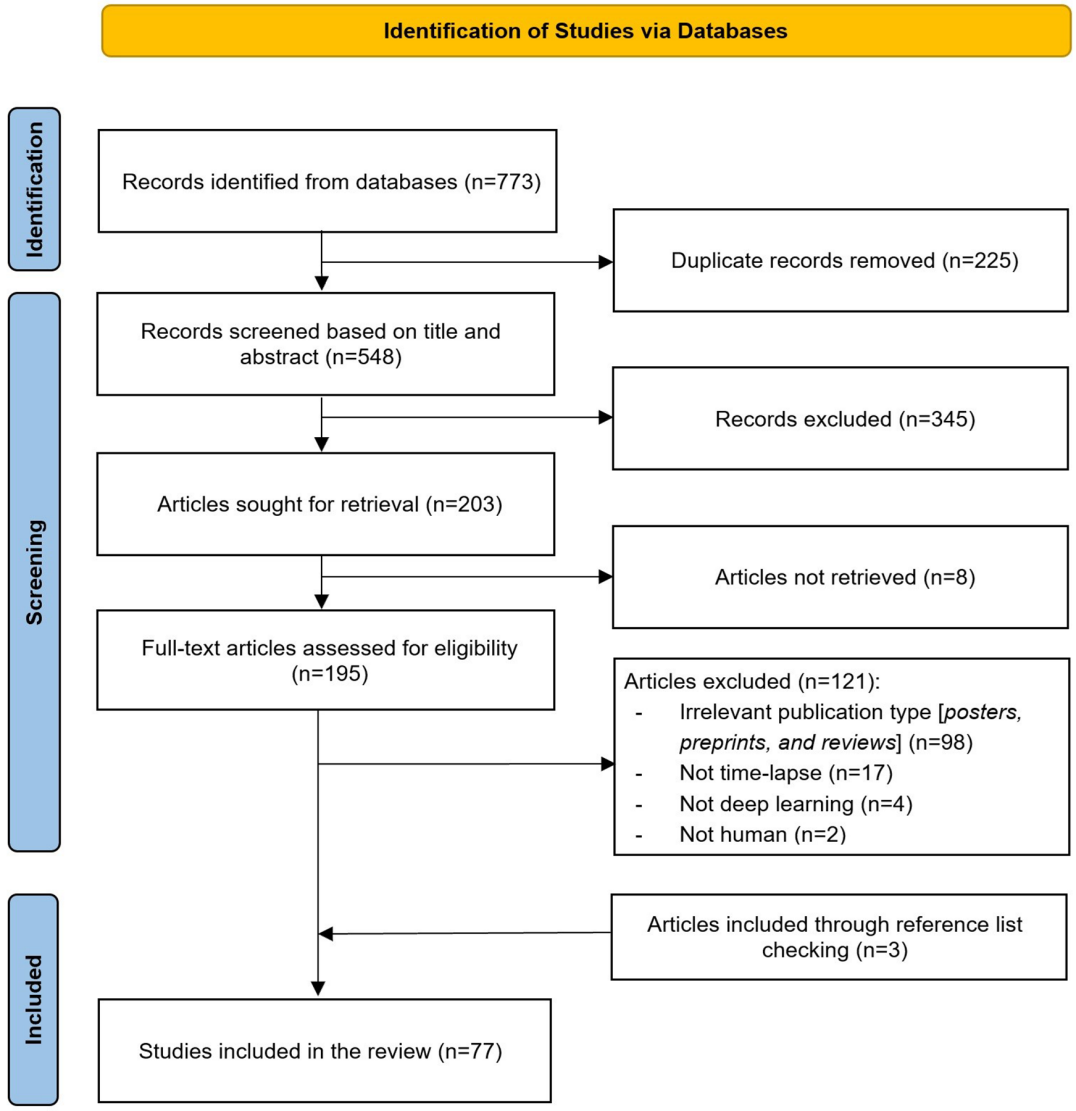
Flow chart of the study selection process. This figure illustrates the step-by-step process of study identification, screening, and inclusion in the review.

Figure 2 illustrates the search results from the pre-selected databases, which initially yielded 773 articles. After identifying and removing 225 duplicates (29%), 548 articles (71%) remained for further review. The titles and abstracts of these remaining articles were screened, leading to the exclusion of 345 articles (45%). Of the remaining 203 records (26%), we were unable to obtain the full text for 8 records (1%). Full-text screening of the remaining 195 articles (25%) led to the exclusion of 121 articles (15%) for various reasons described in Figure 2. Additionally, 3 more articles were identified as relevant through backward and forward referencing, resulting in a total of 77 articles (10%) for inclusion in this review (11, 12, 19–42, 10, 43–48, 7, 49–91).

### 3.2 Characteristics of the included studies

#### 3.2.1 Publication characteristics

**Figure 3:**
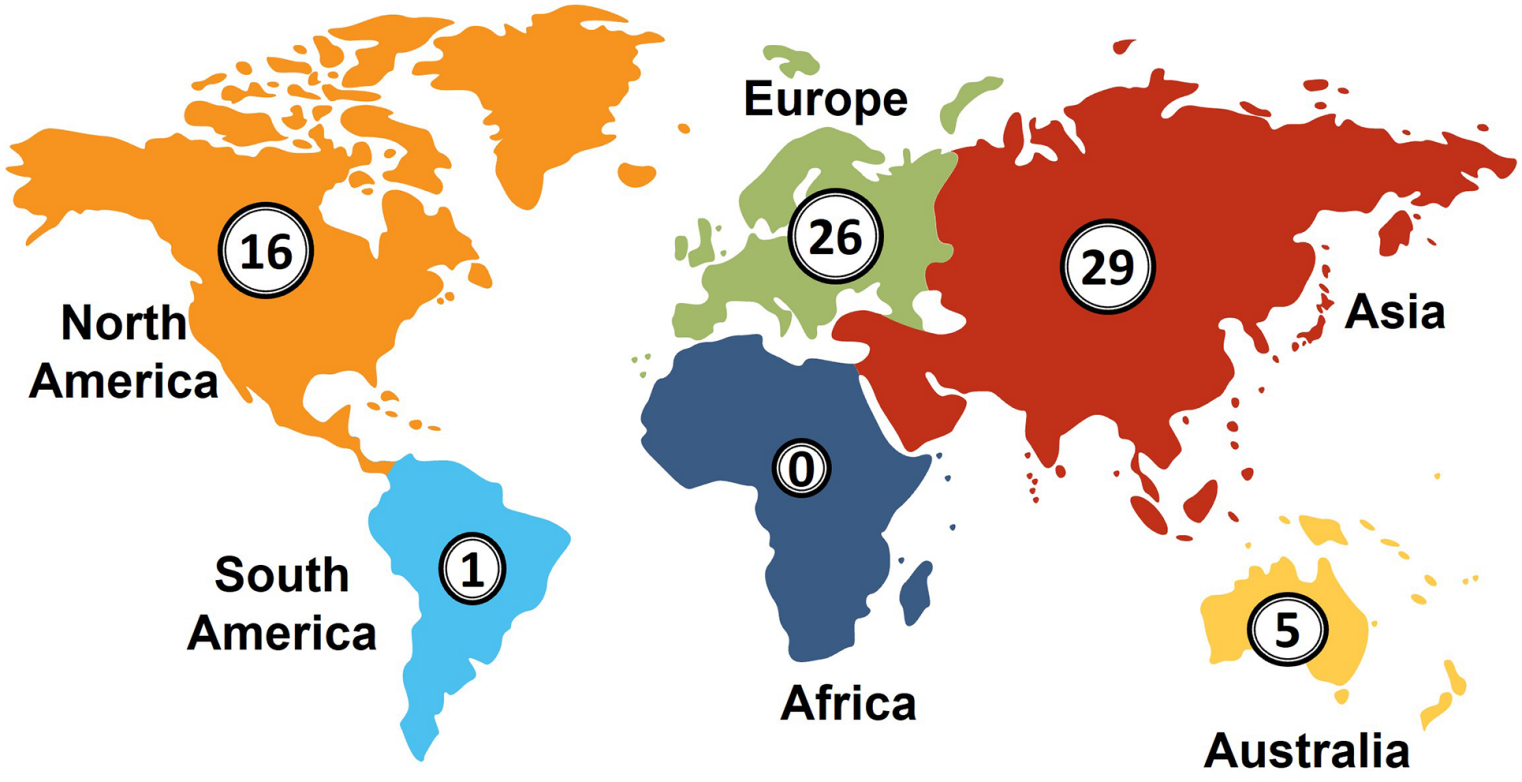
Geographical distribution of included studies. The number inside each circle represents the count of studies from that region. Color coding is used for visual distinction and does not indicate any categorical grouping.

Studies included in this review were published across 20 countries, highlighting the global interest in the application of deep learning and time-lapse imaging in the embryology lab, as shown in Figure 3. Asia leads with 29 studies, constituting 38% of the total, with significant contributions from China (16%, *n* = 13) and Japan (10%, *n* = 8). Europe follows with 26 studies, accounting for 34% of the total, with notable contributions from Denmark (6%, *n* = 5), and Italy and France each contributing 4 studies (5%). North America contributed 16 studies, making up 21% of the total.

As shown in Table 1, the vast majority of the included studies were journal articles, accounting for 82% (*n* = 63) of the publications. Regarding the year of publication, there was a noticeable increase in research output over the last few years. The majority of studies were published in 2023 (29%, *n* = 22) and 2022 (26%, *n* = 20), indicating a recent surge in research activity. For 2024, the number of studies (4%, *n* = 3) reflects only those published between January and May, suggesting that the full year's output may follow the upward trend observed in recent years.

**Table 1:** Characteristics of the included studies.

Feature	Number of studies (%)	Study references
Year of publication		
2024	3 (4%)	(29, 71, 85)
2023	22 (29%)	(12, 21, 22, 30, 35, 40, 42, 47, 50, 53, 61, 65, 69, 70, 74, 78, 80, 81, 84, 86, 88, 90)
2022	20 (26%)	(23, 24, 31, 32, 34, 36–38, 10, 45, 46, 7, 51, 57, 66, 68, 73, 82, 87, 91)
2021	14 (18%)	(11, 19, 20, 25, 27, 33, 48, 49, 59, 60, 64, 77, 83, 89)
2020	5 (6%)	(26, 28, 44, 58, 63)
2019	10 (13%)	(39, 41, 43, 52, 55, 56, 62, 72, 75, 79)
2017	2 (3%)	(67, 76)
2016	1 (1%)	(54)
Type of publication		
Journal article	63 (82%)	(12, 21–35, 37–42, 10, 43–48, 7, 49, 50, 52, 53, 55–57, 59–61, 63, 64, 69, 70, 72, 82, 84, 87)
Conference paper	14 (18%)	(19, 20, 36, 51, 54, 58, 63, 64, 69, 70, 72, 82, 84, 87)
Study type		
Retrospective	72 (94%)	(11, 12, 19–26, 28–33, 35, 36, 39–42, 10, 43–48, 7, 49–91)
Retrospective and prospective	4 (5%)	(27, 37, 38)
Prospective	1 (1%)	(34)
Single/Multi-site		
Single site	36 (47%)	(11, 23, 25, 27, 29, 30, 32, 35, 42, 10, 43, 45, 46, 48, 7, 49, 51, 61, 62, 66, 67, 70, 71, 73, 76, 78, 80, 83, 85, 87–91)
Multi-site	16 (21%)	(12, 21, 22, 24, 26, 28, 37, 38, 40, 41, 50, 55–57, 79, 82)
NR	25 (32%)	(19, 20, 31, 33, 36, 39, 44, 47, 52–54, 58–60, 63, 64, 68, 69, 72, 74, 75, 77, 81, 84, 86)
Number of participants (patients/cycles)		
Mean (Standard Deviation)	2,154 (5,589)	(21–23, 25, 26, 28–32, 35–38, 40, 41, 10, 43, 45, 46, 48, 7, 51, 53, 56, 85–88)
Range	14–34,620	
NR	38 (49%)	(19, 20, 24, 27, 33, 34, 39, 42, 44, 47, 49–52, 55, 57, 58, 60–65, 67, 68, 72, 74–76, 78, 80, 82, 84–88, 90)
Patients’ maternal age		
Mean (standard deviation)	35.7 (3.1)	(24, 25, 28, 10, 53, 56, 60, 66, 69, 71, 79, 81, 89, 90)
Range	18–52	
NR	63 (82%)	(11, 12, 26, 27, 29–42, 43–52, 54, 55, 57–59, 61–65, 67, 68, 70, 72–78, 80, 82–88, 91)
Data sources		
Private	76 (99%)	(11, 12, 19–42, 10, 43–48, 7, 49–58, 65, 67–91)
Public and private	1 (1%)	(82)
Public	0 (0%)	-

NR: Not Reported.

#### 3.2.2 Study type and sites

The vast majority of the included studies (94%, *n* = 72) were retrospective in nature, with a small proportion being a combination of retrospective and prospective (5%, *n* = 4) or solely prospective (1%, *n* = 1). Regarding the study sites (clinics), 47% (*n* = 36) were conducted at a single site, while 21% (*n* = 16) were multi-site studies.

#### 3.2.3 Participants and data sources

The studies varied significantly in terms of the number of participants, with a mean of 2,154 (SD = 5,589) and a range from 14 to 34,620 participants or cycles. However, nearly half of the studies (49%, *n* = 38) did not report the number of participants or cycles. The average maternal age of patients was reported in only 18% (*n* = 14) of the studies, with a mean age of 35.7 years (SD = 3.1). Data sources were predominantly private (99%, *n* = 76), with only one study (1%) utilizing both public and private sources. Supplementary Appendix 4 provides detailed characteristics of each included study.

### 3.3 Applications and outcomes

The primary applications of the studies included monitoring embryo development, assessing and grading embryo quality (61%, *n* = 47), predicting pregnancy and implantation outcomes (35%, *n* = 27), and determining embryo chromosomal composition (31%, *n* = 24). Specific applications ranged from morphologic and morphometric analysis (27%, *n* = 21) and stage classification (26%, *n* = 20) to blastocyst formation and expansion (24%, *n* = 19), blastocyst grading (15%, *n* = 12), and implantation rate prediction (13%, *n* = 10). Outcome measures primarily focused on embryo and blastocyst morphology quality and grading (45%, *n* = 35) and successful IVF outcomes (45%, *n* = 35). The gold standard (ground truth) used in these studies was primarily determined by embryologists (54%, *n* = 42), as shown in Table 2. Supplementary Appendix 5 shows applications and outcomes details in each included study.

**Table 2:** Applications and outcomes of deep learning-assisted embryo assessment.

Feature	Number of studies (%)	Study references
Main applications		
Embryo development/quality assessment/grading	47(61%)	(11, 22, 24, 26, 27, 29–38, 42, 10, 43, 45–48, 49–53, 56, 57, 59–62, 72, 74, 76, 77, 80, 82–87, 89, 91)
Pregnancy/implantation prediction	27(35%)	(12, 19, 20, 23–28, 38, 40, 41, 7, 50, 57, 65, 67, 68, 72, 74, 77, 79, 81, 86, 88, 90, 91)
Embryo chromosomal composition	25(32%)	(21, 27, 29, 33, 34, 39, 10, 43–46, 54, 56, 58, 62–65, 69, 70, 75, 78, 80, 86, 88)
Specific application		
1. Embryo Development/Quality Assessment/Grading a. Morphologic/morphometric	21(27%)	(12, 24, 26, 27, 29–31, 33–36, 42, 43–45, 48, 50, 51, 74, 83)
b. Stage classification	20(26%)	(21, 27, 33, 34, 39, 10, 43, 45, 46, 54, 58, 62–65, 69, 70, 75, 78, 80)
c. Blastocyst formation and expansion	19(24%)	(27, 30–34, 36, 38, 10, 43, 45, 47, 49, 51–53, 57, 61, 83)
d. Blastocyst grading	11(14%)	(30, 37, 38, 43, 50–53, 56, 57, 61, 83)
e. Pronuclear staging/segmentation	4(5%)	(10, 44, 58, 89)
f. Cytoplasm segmentation/ZP segmentation	4(5%)	(58, 80, 82, 89)
g. Usable blastocysts	2(3%)	(43, 60)
2. Pregnancy/Implantation prediction		
a. Implantation rate	9 (12%)	(12, 19, 20, 26, 57, 67, 72, 79, 91)
b. FH pregnancy prediction	10 (13%)	(24, 27, 28, 38, 40, 41, 57, 74, 81, 90)
c. Live-birth prediction	8 (10%)	(23, 25, 7, 65, 68, 77, 86, 88)
3. Embryo chromosomal composition		
a. Ploidy status	4 (5%)	(19, 29, 57, 86, 88)
Outcome measure		
Embryo/blastocyst morphology quality/grading	35 (45%)	(11, 22, 24, 26, 31–38, 42, 10, 46–48, 51, 52, 54–56, 58–61, 66, 73, 76, 82–87, 89)
Successful IVF	35 (45%)	(12, 19–21, 23–28, 30–32, 37, 38, 40, 41, 43, 7, 49, 50, 57, 65, 67, 68, 71, 72, 74, 77, 79, 81, 86, 88, 90, 91)
Ploidy status	4 (5%)	(29, 57, 86, 88)
Reference standard		
Embryologists	42 (54%)	(11, 22, 24, 26, 27, 29, 31–39, 42, 10, 44–48, 51–55, 60, 61, 63, 66, 69, 74, 75, 78, 80, 82–84, 86, 87, 89)
Ultrasound	19 (25%)	(12, 19, 20, 24, 26–28, 37, 38, 40, 41, 43, 50, 56, 57, 67, 79, 90, 91)
Live-birth delivery	14 (18%)	(21, 23, 25, 30–32, 38, 7, 49, 65, 68, 70, 71, 86, 88)
PGT-A	4 (5%)	(29, 57, 86, 88)
NR	11 (14%)	(58, 59, 62, 64, 70, 72, 73, 76, 77, 81, 85)

NR: Not Reported; FH: Fetal Heartbeat; ZP: Zona Pellucida; IVF: *in vitro* Fertilization; PGT-A: Preimplantation Genetic Testing for Aneuploidies.

### 3.4 Embryology and time-lapse characteristics

#### 3.4.1 Embryo population and time-lapse platforms

As shown in Table 3, the number of embryos involved in the studies exhibited significant variation, with a mean of 10,485 (SD = 35,593) and a range from 20 to 249,635 embryos. Additionally, the studies demonstrated considerable diversity in terms of the days of embryo development assessed. The majority of studies focused on the blastocyst stage (47%, *n* = 36), followed by studies on both cleavage and blastocyst stages combined (23%, *n* = 18). Time-lapse technology was predominantly used, with EmbryoScope being the most common (71%, *n* = 55). Other platforms such as Miri (5%, *n* = 4) and GERI (4%, *n* = 3) were less frequently utilized. The interval for time-lapse imaging varied, with the most common interval being 10 min (26%, *n* = 20).

**Table 3:** Embryology and time-lapse characteristics.

Feature	Number of studies (%)	Study references
Number of embryos		
Mean (Standard Deviation)	10,485 (35,593)	(11, 12, 19–35, 40–42, 10, 43–48, 7, 49–51, 53–61, 63–67, 69, 71–75, 77, 79, 80, 87–91)
Range	20–249,635	
NR	16 (21%)	(36–39, 52, 62, 68, 70, 76, 78, 81–86)
Embryo stage		
Pronuclear stage (D1)	1 (1%)	(89)
Cleavage stage (D1- D4)	5 (6%)	(21, 29, 67, 71, 90)
Blastocyst stage (D5- D7)	36 (47%)	(20, 22, 24, 30–32, 35, 37, 38, 41, 10, 43, 45, 47–53, 56, 57, 59–61, 65, 74, 75, 79, 81, 82, 84, 85, 88, 91)
Cleavage and blastocyst	18 (23%)	(12, 19, 25, 27, 33, 40, 44, 46, 54, 58, 64, 70, 72, 73, 77, 78, 80, 86)
NR	17 (22%)	(11, 23, 26, 28, 34, 36, 39, 42, 55, 62, 63, 66, 68, 69, 76, 83, 87)
Time-lapse technology used		
EmbryoScope	55 (71%)	(11, 12, 19–33, 41, 42, 10, 43, 45–48, 7, 49, 52, 53, 55–59, 61, 62, 64–67, 71, 73–79, 81–83, 87, 89–91)
Miri	4 (5%)	(34, 35, 39, 75)
GERI	3 (4%)	(37, 38, 40)
Others (Primo Vision, Eeva, Olympus IX71)	3 (4%)	(54, 82, 86)
NR	14 (18%)	(36, 44, 50, 51, 60, 63, 68–70, 72, 80, 84, 85, 88)
Time-lapse interval (minutes)		
5	3 (4%)	(39, 54, 75)
7	1 (1%)	(67)
10	20 (26%)	(11, 12, 21, 22, 27, 29, 41, 10, 43, 46, 56, 57, 62, 68, 71, 73, 77, 86, 87, 89)
15	12 (16%)	(19, 20, 24, 29, 33, 56, 57, 63, 68, 69, 77, 87, 91)
20	4 (5%)	(55, 58, 64, 73)
30	2 (3%)	(12, 74)
NR	41 (53%)	(23, 25, 26, 28, 30–32, 34–38, 40, 42, 44, 47, 48, 7, 49–53, 59–61, 65, 66, 70, 72, 76, 78–85, 88, 90)
Annotation standard		
Gardner	22 (28%)	(22, 24, 30–32, 37, 38, 40–42, 10, 43, 53, 55–57, 65, 71, 72, 77, 81, 88)
ASEBIR	4 (5%)	(25, 27, 40, 73)
Alpha ESHRE Consensus	2 (3%)	(21, 33)
NR	49 (63%)	(11, 12, 19, 20, 23, 26, 28, 29, 34–36, 39, 44–48, 7, 49–52, 54, 58–60, 62–64, 66–70, 72, 74–76, 78–80, 82–87, 89–91)
Commercial software		
iDAScore	13 (16%)	(12, 21, 24, 30, 10, 43, 50, 53, 57, 59, 71, 81, 90)
KIDScore	3 (4%)	(27, 74, 88)
CHLOETM	2 (3%)	(31, 32)
IVY	2 (3%)	(41, 79)
CNTK	1 (1%)	(44)
No software reported	57 (74%)	(11, 19, 20, 22, 23, 25, 26, 29, 33–40, 42, 45–48, 7, 49, 51, 52, 54–56, 58, 60–70, 72–78, 80, 82–87, 89, 91)

NR: Not Reported; D1: Day 1; D4: Day 4; D5: Day 5; D7: Day 7.

#### 3.4.2 Annotation standards

The annotation standards used in the studies also varied, with the Gardner criteria being the most frequently applied (28%, *n* = 22). However, more than half of the studies (63%, *n* = 49) did not report the annotation standard used. Regarding commercial software, iDAScore was the most commonly used, appearing in 16% (*n* = 13) of the studies. Notably, 73% (*n* = 58) of the studies did not use any commercial software. Supplementary Appendix 6 includes embryology and time-lapse characteristics in each included study.

### 3.5 Deep learning models characteristics

#### 3.5.1 Deep learning architectures

**Figure 4:**
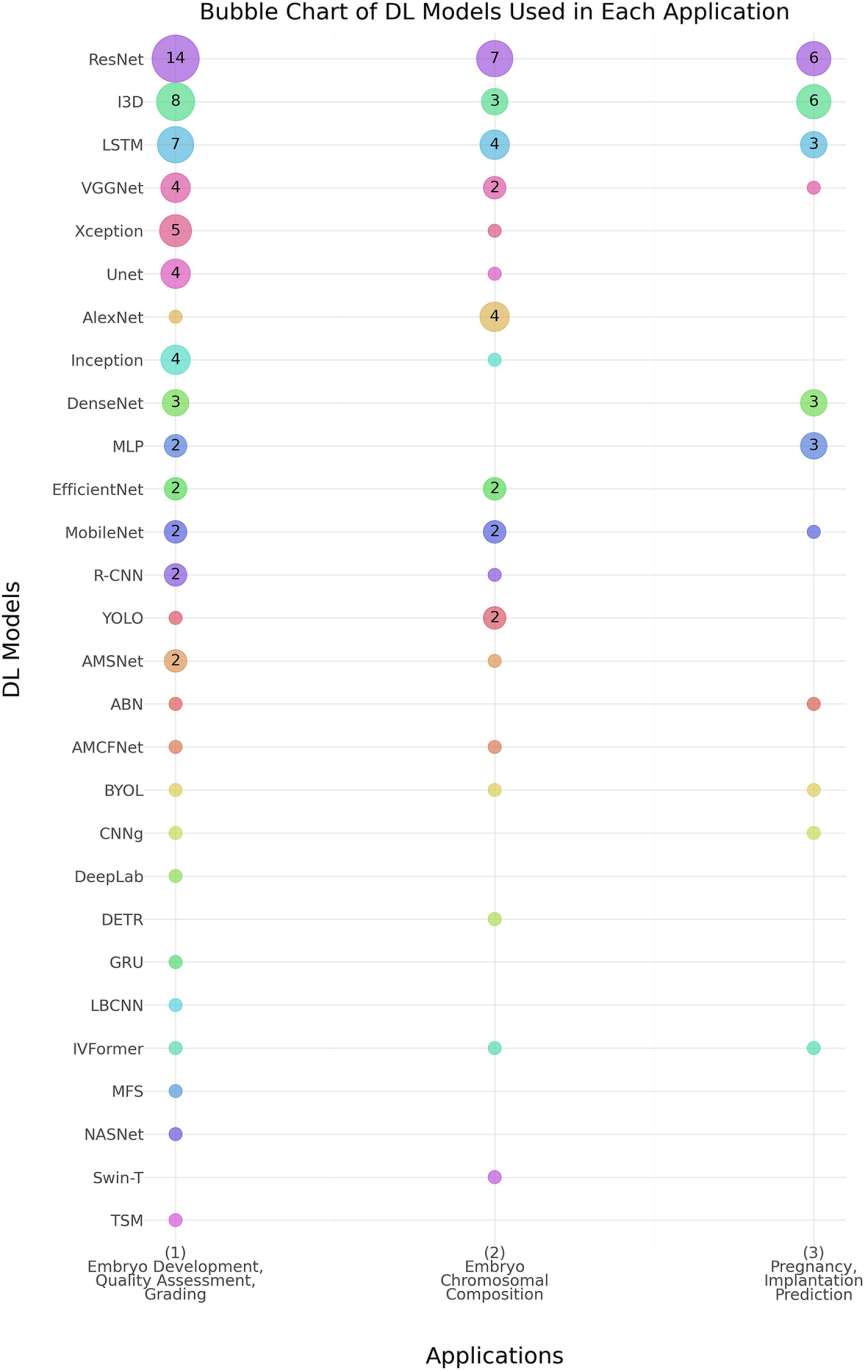
Distribution of deep learning (DL) models across Various embryo assessment applications. The chart illustrates the prevalence of different DL models used in three key applications: (1) Embryo Development, Quality Assessment, and Grading, (2) Embryo Chromosomal Composition, and (3) Pregnancy & Implantation Prediction. Numbers inside the bubbles represent the number of studies. Bubbles without numbers indicate a count of 1 study. Abbreviations- ABN, Attention-Based Network; AMCFNet, Attention Mechanism Convolutional Fusion Networks; AMSNet, Adaptive Multi-Scale Network; BYOL, Bootstrap Your Own Latent; CNN, Convolutional Neural Network; CNNg, CNN + Genetic Algorithm; DeepLab, Deep Labelling; DenseNet, Densely Connected Convolutional Networks; DETR, Detection Transformer; DNN, Deep Neural Network; EfficientNet, Efficient Neural Network; GRU, Gated Recurrent Unit; I3D, Inflated 3D Convolutional Network; Inception, Inception Network; IVFormer, Intermediate Visual Transformer; LBCNN, Learned Binary Convolutional Neural Network; LSTM, Long Short-Term Memory; MLP, Multi-Layer Perceptron; MobileNet, Mobile Neural Network; NASNet, Neural Architecture Search Network; R-CNN, Regions with Convolutional Neural Networks; ResNet, Residual Network; RNN, Recurrent Neural Network; Swin-T, Swin Transformer; TSM, Temporal Shift Module; Unet, U-Net Convolutional Network; VGGNet, Visual Geometry Group Network; Xception, Extreme Inception; YOLO, You Only Look Once.

As shown in Table 4, the predominant deep learning architecture used in the studies was convolutional neural networks (CNNs), which accounted for 81% (*n* = 62) of the studies. Recurrent neural networks (RNNs) were employed in 16% (*n* = 10) of the studies. Figure 4 shows the prevalence of different DL models used in the three main applications. ResNet, I3D, and LSTM models show higher usage, especially in embryo development and quality assessment, indicating a preference for these models in this application area.

**Table 4:** Deep learning models characteristics.

Feature	Number of studies (%)	Study references
Input training data		
Image features	77 (100%)	(11, 12, 19–42, 10, 43–48, 7, 49–91)
Demographics	11 (14%)	(12, 22, 23, 29, 37, 40, 48, 67, 74, 88, 91)
IVF cycle parameters	5 (6%)	(22, 23, 40, 88, 91)
Clinical and reproductive history	4 (5%)	(22, 29, 40, 90)
Male data	3 (4%)	(22, 40, 91)
Main deep learning architecture		
CNNs	62 (81%)	(11, 12, 19–21, 24, 26, 29, 30, 34–40, 42, 10, 43, 45, 46, 48, 7, 49–63, 65, 66, 68–75, 77, 78, 80–90)
RNNs	10 (16%)	(23, 33, 42, 45, 56, 60, 64, 74, 83, 91)
DNNs	9 (12%)	(22, 23, 25, 28, 41, 47, 67, 76, 79)
Transformers	3 (4%)	(70, 78, 86)
NR	4 (5%)	(27, 31, 32, 44)
Specific deep learning architecture		
ResNet	21 (27%)	(11, 20, 34, 36–38, 40, 45, 46, 48, 7, 51, 58, 61, 62, 64, 66, 68, 69, 73, 87)
I3D	13 (17%)	(12, 21, 24, 30, 10, 43, 50, 53, 57, 59, 71, 81, 90)
LSTM	10 (13%)	(23, 30, 42, 45, 56, 60, 64, 74, 83, 91)
Unet	5 (6%)	(36, 49, 51, 63, 82)
VGGNet	6 (8%)	(35, 73–75, 83, 84)
AlexNet	5 (6%)	(39, 62, 70, 75, 84)
Xception	5 (6%)	(11, 46, 52, 56, 83)
DenseNet	4 (5%)	(35, 60, 66, 83)
MLP	5 (6%)	(22, 23, 25, 67, 73)
Unspecified CNN	5 (6%)	(19, 54, 72, 88, 89)
Unspecified DNN	5 (6%)	(28, 41, 47, 76, 79)
EfficientNet	3 (4%)	(34, 51, 70)
Inception	3 (4%)	(34, 55, 83)
MobileNet	3 (4%)	(34, 65, 83)
R-CNN	2 (3%)	(37, 58)
YOLO	2 (3%)	(78, 80)
AMSNet	2 (3%)	(29, 87)
Others (ABN, AMCFNet, BYOL, CNNg, DeepLab, DETR, GRU, LBCNN, IVFormer, MFS,NASNet, Swin-T, TSM)	14 (18%)	(11, 26, 28, 41, 42, 47, 70, 76–79, 83, 85, 86)
Validation method		
Hold-out	48 (62%)	(11, 12, 21, 25, 27, 29, 30, 34–36, 38, 39, 41, 42, 10, 44, 7, 49, 50, 52, 53, 55, 56, 58–62, 64–67, 70–73, 76, 78–81, 83–85, 87, 88, 90)
K-fold	31 (40%)	(19, 20, 22–24, 28, 31–33, 37, 40, 43, 45–48, 7, 51, 54, 57, 63, 68, 69, 74, 75, 77, 79, 82, 86, 89, 91)
Performance metric		
ACC	45 (58%)	(11, 19, 20, 22, 23, 33–40, 42, 44–46, 48, 51, 52, 54–56, 58, 60–65, 69, 70, 72–76, 78, 80, 82–84, 87, 89, 91)
AUC-ROC	44 (57%)	(11, 12, 21–30, 37, 38, 40, 42, 48, 7, 50–53, 55, 57, 59–61, 65–69, 71, 73, 74, 77, 79, 81, 86–88, 90, 91)
PREC	20 (26%)	(19, 20, 22, 23, 33, 35, 36, 42, 58, 59, 68, 69, 73, 75, 78, 80, 82, 84, 89, 91)
REC	20 (26%)	(19, 20, 22, 23, 33, 35, 36, 42, 58, 59, 68, 69, 73, 75, 78, 80, 82, 84, 89, 91)
SENS	9 (12%)	(22, 33, 37, 40, 44, 70, 72, 73, 78)
F1	9 (12%)	(22, 23, 33, 35, 42, 73, 78, 84, 91)
*p*-value	9 (12%)	(31, 32, 41, 10, 43, 49, 71, 85, 88)
SPES	8 (10%)	(11, 22, 33, 70, 72, 73, 78, 86)
Jaccard-Index	3 (4%)	(19, 20, 82)
MSE	3 (4%)	(62, 83)
Others (AUPRC, FPR, NPV, MCC, Dice, r, PPV, MAE)	8 (10%)	(22, 45, 68, 70, 74, 78, 82, 91)
NR	1 (1%)	(47)

ABN, Attention-Based Network; ACC, Accuracy; AMCFNet, Attention Mechanism Convolutional Fusion Networks; AMSNet, Adaptive Multi-Scale Network; AUC-ROC, Area Under the Curve - Receiver Operating Characteristic; AUPRC, Area Under the Precision Recall Curve; BYOL, Bootstrap Your Own Latent; CNN, Convolutional Neural Network; CNNg, CNN + Genetic Algorithm; DeepLab, Deep Labelling; DenseNet, Densely Connected Convolutional Networks; DETR, Detection Transformer; Dice, Dice coefficient; DNN, Deep Neural Network; EfficientNet, Efficient Neural Network; F1, F Score; FPR, False Positive Rate; GRU, Gated Recurrent Unit; I3D, Inflated 3D Convolutional Network; Inception, Inception Network; IVF, *in vitro* Fertilization; IVFormer, Intermediate Visual Transformer; Jaccard-Index, Jaccard similarity coefficient; LBCNN, Learned Binary Convolutional Neural Network; LSTM, Long Short-Term Memory; MAE, Mean Absolute Error; MCC, Matthews Correlation Coefficient; MFS, Multi-Frequency Series; MLP, Multi-Layer Perceptron; MobileNet, Mobile Neural Network; MSE, Mean Squared Error; NASNet, Neural Architecture Search Network; NR, Not Reported; NPV, Negative Predictive Value; PPV, Positive Predictive Value; PREC, Precision; R-CNN, Regions with Convolutional Neural Networks; R, Correlation Coefficient; REC, Recall; ResNet, Residual Network; RNN, Recurrent Neural Network; SENS, Sensitivity; SPES, Specificity; Swin-T, Swin Transformer; TSM, Temporal Shift Module; Unet, U-Net Convolutional Network; VGGNet, Visual Geometry Group Network; Xception, Extreme Inception; YOLO, You Only Look Once.

#### 3.5.2 Input data types

All studies used video image features as training data (100%, *n* = 77). Additionally, some studies incorporated demographics (14%, *n* = 11) and IVF cycle parameters (6%, *n* = 6). A small number of studies (3%, *n* = 2) utilized a more comprehensive dataset that included image features, demographics, clinical and reproductive history, IVF cycle parameters, and male data (Table 5).

**Table 5:** Data types used by the included studies.

Data type	Number of studies (%)	Study references
TL image features	77 (100%)	(11, 12, 19–48, 7, 49–91)
TL image features + clinical and reproductive history	1 (1%)	(90)
TL image features + demographics	5 (5%)	(12, 37, 48, 67, 74)
TL image features + demographics + clinical and reproductive history	1 (1%)	(29)
TL image features + demographics + IVF cycle data	2 (3%)	(23, 88)
TL image features + demographics + IVF cycle data + male partner data	1 (1%)	(91)
TL image features + demographics + IVF cycle data + male partner data + clinical and reproductive history	2 (3%)	(22, 40)

IVF, *in vitro* Fertilization; TL, Time-lapse.

#### 3.5.3 Validation methods and performance metrics

For validation of the deep learning models, the hold-out method was the most common, used in 62% (*n* = 48) of the studies. The performance of the deep learning models was evaluated using various metrics. Accuracy (ACC) was the most commonly reported metric, used in 58% (*n* = 45) of the studies. Additionally, the area under the receiver operating characteristic curve (AUC-ROC) was used in 57% (*n* = 44) of the studies. Supplementary Appendix 7 shows deep learning models characteristics in each included study.

## 4 Discussion

### 4.1 Main findings

In this scoping review, we aim to provide a focused and comprehensive analysis of the application of deep learning and time-lapse imaging in embryo assessment. Specifically, we investigate the characteristics of deep learning models used for evaluating and selecting embryos monitored through time-lapse imaging, examining the characteristics of the included studies, target applications, outcomes, and features of embryology and time-lapse platforms.

The field of DL-powered embryo imaging research is relatively recent and has experienced steady growth, with publications increasing approximately fourfold from 2020 to 2023. Interestingly, there was a decline in the number of studies from 2019 to 2020, possibly due to the disruptions caused by the COVID-19 pandemic. However, the subsequent years saw a significant increase, highlighting the growing recognition and interest in this field.

Most studies originate from high-income countries (70%, 54 studies), using datasets from top-tier laboratories equipped with time-lapse incubators, providing what is considered the ideal dataset (optimal lab conditions, culture systems, and embryo transfer practitioners). These ideal conditions allow for effective testing of deep learning models for outcome prediction. However, in the real world, not all labs are equipped with time-lapse incubators, and many other factors influence outcomes. This poses challenges to the generalizability of the results. Therefore, datasets should reflect variations in patient demographics and IVF success determinants to improve applicability. The data used in DL models range from tens to hundreds of thousands of cycles or patients, with larger datasets generally providing more reliable results. However, a significant amount of unreported data, particularly concerning patients' maternal age (82%, 63 studies), and the use of single-center datasets (47%, 36 studies) are limitations of these studies. Additionally, most studies use private datasets (99%), restricting reproducibility. These factors limit the generalizability of the models and hinder systematic evaluation of robustness and potential biases. Biases in these datasets stem from multiple sources, including demographic homogeneity, clinical practice variations, and data preprocessing inconsistencies. For example, models trained predominantly on datasets from high-income countries may not generalize well to lower-resource settings where laboratory conditions and patient populations differ significantly. Moreover, patient selection bias—where certain demographic groups are underrepresented—can lead to disparities in model performance. Algorithmic biases may also emerge from skewed labeling practices or feature imbalances, such as reliance on clinical parameters that do not equally affect all patient subgroups. To mitigate these biases, several strategies should be employed. First, diversifying datasets by integrating data from multiple geographic regions, clinical settings, and patient backgrounds can enhance model robustness. Second, implementing bias detection techniques, such as fairnessaware machine learning frameworks, can systematically assess and quantify disparities in model performance across different subgroups. Finally, explainable AI (XAI) methods should be leveraged to identify and correct sources of bias, ensuring that predictions align with clinically relevant factors rather than spurious correlations.

The majority of publications on the use of DL-powered TLI models in ART focused on embryo assessment (61%, 47 studies). These studies show promise in supporting embryologists' decisionmaking for ranking or selecting embryos for cryopreservation or transfer. However, only 35% (27 studies) used deep learning models for predicting pregnancy and implantation, which are the ultimate goals of IVF treatments. For instance, 8 studies (10%) applied DL models for predicting live birth. This focus on short-term endpoints in the literature, due to the accessibility of data, paves the way for future automation of IVF laboratories. Moreover, developing associations and prediction models for clinical outcomes is increasingly complex, involving not only embryo viability but also various implantation-related factors related to female biology.

The amount of data used for developing the DL models varies widely, ranging from tens to hundreds of thousands of embryos. Some teams included images from both cleavage and blastocyst stage embryos (23%, 18 studies), which is beneficial for constructing robust models. In contrast, nearly half of the literature (47%, 36 studies) relied solely on blastocyst images.

Excluding cleavage stage embryos, which in many cases can arrest before reaching the blastocyst stage, from the training data can make the models less robust. This exclusion causes the model to miss out on critical information about early developmental failures and the deselection of embryos, reducing the diversity of the dataset. The model can be generalized if the duration of incubation related to embryo development is included. Most of the literature used the time-lapse EmbryoScope incubator platform (71%, 55 studies) as it was the first commercial time-lapse incubator introduced to the market. This platform's higher data availability and accessibility, along with its larger market share and greater R&D investment, explain its prevalence in the literature. Additionally, 16% (13 studies) of the literature relied on commercially available software algorithms such as iDAScore, a ranking-based tool used to predict the likelihood of a fetal heartbeat. It is important to reflect that deep learning methods, including commercially available tools that employed multisite datasets, can still be considered “black boxes”. This is because their interpretations are not transparent and are often based solely on rankings. Notably, most of the literature did not rely on commercially available software (74%, 57 studies). This suggests that DL research in ART is still highly experimental and evolving. Researchers are often developing their own algorithms and methodologies, tailored to their specific datasets and research objectives, rather than relying solely on pre-existing commercial solutions.

Convolutional neural networks (CNNs) were widely employed in 81% of the studies for the analysis of embryonic imaging data. Unlike regular neural networks, CNNs have neurons arranged in three dimensions: width, height, and depth, enabling them to effectively capture spatial hierarchies in images. Recently, advanced architectures of CNNs, such as residual networks (ResNet) (92) and Inception (93), have significantly accelerated the progress of deep learning methods in image classification, providing enhanced accuracy and robustness. These deep architectures allow for more efficient processing of complex visual data, making them particularly well-suited for detailed analysis required in IVF embryology. Despite CNNs being the predominant choice, alternative deep learning architectures have been explored to address specific challenges in embryo assessment. Recurrent neural networks (RNNs), particularly Long ShortTerm Memory (LSTM) models, have been applied to analyze sequential embryo development data, leveraging their ability to capture temporal dependencies in time-lapse imaging. Transformerbased models, such as Vision Transformers (ViTs) and Swin Transformers, have recently emerged as promising alternatives due to their capability to model long-range dependencies more effectively than CNNs. Studies suggest that ViTs may outperform traditional CNNs in some medical imaging applications by capturing global contextual information rather than relying solely on localized features. However, their application in embryo assessment remains limited, likely due to the high data requirements and computational cost associated with training transformer models.

An important decision related to the evaluation of the deep learning models is the selection of the data split strategy. Nearly half of the studies used the hold-out method for cross-validation, likely due to its simplicity and computational efficiency. Hold-out method requires less computational effort than k-fold cross-validation and provides a consistent benchmark for model comparison, making it easier to replicate results.

The evaluation of deep learning models for embryo selection is crucial for assessing their effectiveness, reliability, and generalizability. Various metrics are employed in the reviewed studies to evaluate performance. Accuracy, the most commonly used metric, measures the proportion of correct predictions and is easily understood. However, it should be used cautiously with unbalanced datasets. Therefore, it is essential to choose appropriate metrics, such as AUCROC, precision, recall, or F1-score, to better evaluate performance in such cases.

### 4.2 Research and clinical implications

#### 4.2.1 Need for personalized AI models for embryology outcomes

Only a small fraction (2 out of 77 studies) utilized a richer dataset, which included not only embryo images but also demographics, clinical and reproductive history, IVF cycle parameters, and male data. This limited use underscores the need for more research incorporating comprehensive patient information, such as genetic profiles, lifestyle factors, and environmental influences. By moving towards more personalized AI models for predicting embryo viability and outcomes, we could significantly enhance the accuracy, relevance, and clinical utility of AI-assisted reproductive technologies, ultimately improving patient-specific treatment strategies and success rates in IVF procedures.

#### 4.2.2 Collaboration for public datasets

Notably, 99% of the reviewed studies relied on private datasets, and only 21% leveraged data from multiple clinics. This underscores the urgent need for collaboration to create public, multi-site datasets. - The reliance on private datasets presents several key challenges, including the lack of external validation, limited reproducibility, and potential biases introduced by dataset homogeneity. The limited availability of public datasets hinders the ability to benchmark deep learning models against a standardized dataset, making it difficult to assess their true clinical applicability across diverse populations. Additionally, private datasets often restrict external researchers from accessing raw data, reducing opportunities for independent validation and cross-institutional studies. To address these concerns, there is a growing need for global initiatives to establish public IVF datasets that aggregate diverse patient populations, embryology lab conditions, and clinical outcomes. Encouraging data-sharing agreements among clinics, implementing privacy-preserving techniques such as federated learning, and developing standardized annotation protocols could help mitigate these limitations. By expanding access to high-quality, diverse training datasets, future AI models can achieve improved generalizability, facilitating safer and more equitable AI-driven embryo assessment.

#### 4.2.3 Overcoming deep learning challenges with large language models

Deep learning models trained on high-resolution time-lapse imaging data often struggle to adapt to less expensive, portable optical systems, particularly when data quality is reduced (94). Additionally, CNNs require large, annotated datasets from the target domain, which are challenging to generate, especially for medical devices and newer low-cost, low-resolution hardware. The evolution of large language models (LLMs) can address these challenges in creating automated, accurate, and cost-effective systems for embryo assessments. LLMs can generate synthetic datasets to supplement limited annotated data, enabling effective training of deep learning models even with low-resolution images. Specifically, visual LLMs (95, 96) have the potential to improve the processing and interpretation of embryo images, providing accurate results despite variations in image quality. Moreover, multimodal LLMs (97) can improve domain adaptation by learning from diverse data types, including textual patient clinical and reproductive history, ultrasound images of the ovaries and uterus, TLI embryo images, lab results, and other modalities. This multimodal approach has the potential to enhance the robustness of AI models across various embryology applications and data qualities.

#### 4.2.4 Challenges in integrating AI models into clinical IVF workflows

Despite promising advancements, integrating AI-powered models into routine IVF clinical workflows presents multiple challenges. One primary concern is the lack of standardized protocols for incorporating AI-driven embryo assessments into embryologists' decision-making processes. While AI models can assist in ranking embryos based on viability, their acceptance in clinical settings depends on their interpretability, reliability, and compatibility with existing workflows. Many embryologists remain cautious about adopting AI models due to concerns about automation replacing clinical expertise, especially given that embryo selection is a nuanced process that considers factors beyond image-based assessments. Furthermore, AI models need to be seamlessly integrated into electronic medical record (EMR) systems and IVF lab software to ensure smooth data exchange without disrupting current workflows. The interoperability of AI tools with different embryology platforms remains a technical challenge. Addressing these issues requires collaboration between AI developers, clinicians, and embryology software vendors to ensure that AI-driven embryo assessments complement, rather than replace, clinical judgment. Pilot studies evaluating AI-assisted decision-making in real-world IVF labs are crucial to refining integration strategies and understanding the impact on workflow efficiency.

#### 4.2.5 The importance of model transparency and interpretability

One of the most pressing challenges in AI-driven embryo selection is the issue of model transparency. Deep learning models, particularly convolutional neural networks (CNNs), are often considered “black boxes,” as their decision-making processes are not easily interpretable. This lack of transparency raises concerns about trust, reproducibility, and accountability in clinical decision-making. To address this, explainable AI (XAI) techniques should be employed to make model predictions more interpretable to clinicians. Methods such as Grad-CAM (Gradient-weighted Class Activation Mapping) and SHAP (Shapley Additive Explanations) can help visualize which features in embryo images contribute most to AI predictions. Increasing model transparency will be critical for widespread clinical adoption.

#### 4.2.6 Ethical considerations in AI-assisted embryo selection

Embryo selection is an inherently sensitive process with significant ethical and societal implications. AI-driven ranking systems must not inadvertently prioritize certain embryos based on implicit biases within the training data. Ensuring fairness requires rigorous bias detection and correction measures during model development. AI models should also be continuously monitored for unintended biases, with diverse patient populations included in validation studies. Another ethical concern relates to informed consent and patient autonomy. IVF patients should be made aware of how AI contributes to embryo selection and should retain the right to make final decisions in consultation with their clinicians. Transparency in AI recommendations and clear communication between providers and patients are essential to maintaining ethical standards in AI-driven reproductive medicine.

### 4.3 Limitations

This review has several limitations. First, it excluded studies that used traditional machine learning algorithms, focusing solely on those employing more sophisticated AI algorithms, specifically deep learning. Second, studies that utilized static microscopy images were excluded, with the review concentrating only on those employing time-lapse imaging. Additionally, only studies published in English were included, potentially overlooking relevant research in other languages. Finally, as a scoping review, it did not aim to evaluate the performance of the deep learning methods included. A subsequent systematic review with meta-analysis could assess the effectiveness of these models, potentially by application, time-lapse platforms, training data types, or other factors.

## 5 Conclusion

In conclusion, this scoping review provides a detailed and comprehensive analysis of the application of deep learning and time-lapse imaging in IVF embryo assessment. Our analysis included the target applications, outcomes, features of embryology and time-lapse platforms, and the specifics of the deep learning model architectures employed. By synthesizing these elements, we offer an in-depth understanding of the current state and future potential of AI applications in embryo evaluation and selection. Despite the progress made, significant challenges remain in developing AI models that are both generalizable and clinically robust. Future advancements should prioritize the integration of diverse and multi-institutional datasets to enhance model reliability across different populations and laboratory settings. The development of personalized AI models incorporating patient demographics, genetic factors, and lifestyle parameters will further improve predictive accuracy and clinical utility. Additionally, emerging multimodal AI approaches, including large language models (LLMs), hold promise for improving domain adaptation, enabling models to effectively integrate text-based patient data with embryo imaging for more comprehensive decision support. The reliance on private datasets remains a major limitation, restricting reproducibility, external validation, and broader clinical applicability. To address this, fostering global collaborations to create public, high-quality, and diverse IVF datasets is essential. Implementing privacy-preserving techniques, such as federated learning, and establishing standardized data-sharing agreements among clinics can help overcome data accessibility barriers while maintaining patient confidentiality. Ultimately, expanding AI applications beyond traditional embryo selection—toward predicting broader reproductive outcomes, assessing long-term neonatal health, and integrating AI models into real-world clinical workflows—will be crucial for the next generation of AI-powered IVF solutions. By addressing these challenges, future research can drive more interpretable and clinically impactful AI technologies in reproductive medicine.

## Data Availability

The original contributions presented in the study are included in the article/Supplementary Material, further inquiries can be directed to the corresponding author/s.

## Glossary

ABN: Attention-Based Network
ACC: accuracy
AMCFNet: Attention Mechanism Convolutional Fusion Networks
AMSNet: Adaptive Multi-Scale Network
ART: assisted reproductive technology
AUC-ROC: area under the curve - receiver operating characteristic
AUPRC: area under the precision recall curve
BYOL: bootstrap your own latent
CNN: Convolutional Neural Network
CNNg: CNN + Genetic Algorithm
DeepLab: deep labelling
DenseNet: Densely Connected Convolutional Networks
DETR: detection transformer
Dice: dice coefficient
DNN: Deep Neural Network
EfficientNet: Efficient Neural Network
F1: F score
FPR: false positive rate
GRU: gated recurrent unit
I3D: Inflated 3D Convolutional Network
Inception: Inception Network
IVF: *in vitro* Fertilization
IVFormer: intermediate visual transformer
Jaccard-Index: Jaccard Similarity Coefficient
LBCNN: Learned Binary Convolutional Neural Network
LSTM: long short-term memory
MAE: Mean Absolute Error
MCC: Matthews Correlation Coefficient MFS: Multi-Frequency Series MLP: Multi-Layer Perceptron
MobileNet: Mobile Neural Network
MSE: Mean Squared Error
NASNet: Neural Architecture Search Network
NR: not reported
NPV: negative predictive value
PPV: positive predictive value
PREC: precision
R-CNN: Regions with Convolutional Neural Networks
R: correlation coefficient
REC: recall
ResNet: Residual Network
RNN: Recurrent Neural Network
SENS: sensitivity
SPES: specificity
Swin-T: Swin Transformer
TLI: time-lapse imaging
TSM: Temporal Shift Module Unet: U-Net Convolutional Network
VGGNet: Visual Geometry Group Network
Xception: extreme inception
YOLO: you only look once.
