# Supplementary material for: Deep learning applications for human embryo assessment using time-lapse imaging: scoping review
Source: Front Reprod Health. 2025 Apr 8;7:1549642. doi: 10.3389/frph.2025.1549642 (PMC12011738; doi:10.3389/frph.2025.1549642)
Supplement: Supplementary file 2 [file Table2.docx]

**Multimedia Appendix 2: Search strategy**

Database(s): **Ovid MEDLINE(R) ALL**1946 to November 10, 2023
Search Strategy:

| **#** | **Searches** | **Results** |
| --- | --- | --- |
| 1 | exp Deep Learning/ | 17154 |
| 2 | "Deep Learning".tw. | 47316 |
| 3 | exp Artificial Intelligence/ | 182677 |
| 4 | "Artificial Intelligence".tw. | 34822 |
| 5 | exp Machine Learning/ | 61693 |
| 6 | "Machine Learning".tw. | 86806 |
| 7 | "Convolutional Neural Network*".tw. | 23869 |
| 8 | "Recurrent Neural Network*".tw. | 4223 |
| 9 | "Deep Neural Network*".tw. | 9316 |
| 10 | "Artificial Neural Network*".tw. | 17370 |
| 11 | "Deep Belief Network*".tw. | 422 |
| 12 | "Autoencoder*".tw. | 3086 |
| 13 | "Generative Adversarial Network*".tw. | 3084 |
| 14 | "Long Short-Term Memory Network*".tw. | 654 |
| 15 | "Computer Vision".tw. | 7475 |
| 16 | VGGNet.tw. | 162 |
| 17 | ResNet.tw. | 2538 |
| 18 | exp Time-Lapse Imaging/ | 3558 |
| 19 | "Time-Lapse*".mp. [mp=title, book title, abstract, original title, name of substance word, subject heading word, floating sub-heading word, keyword heading word, organism supplementary concept word, protocol supplementary concept word, rare disease supplementary concept word, unique identifier, synonyms, population supplementary concept word, anatomy supplementary concept word] | 15889 |
| 20 | exp Fertilization in Vitro/ | 41376 |
| 21 | "Fertilization in Vitro".tw. | 730 |
| 22 | "IVF".tw. | 28725 |
| 23 | "ICSI".tw. | 10469 |
| 24 | "Assisted Reproductive Techn*".tw. | 12084 |
| 25 | "Embryo*".tw. | 397212 |
| 26 | "Blastocyst*".tw. | 26772 |
| 27 | 1 or 2 or 3 or 4 or 5 or 6 or 7 or 8 or 9 or 10 or 11 or 12 or 13 or 14 or 15 or 16 or 17 | 280396 |
| 28 | 20 or 21 or 22 or 23 or 24 or 25 or 26 | 434054 |
| 29 | 18 or 19 | 15889 |
| 30 | 27 and 28 and 29 | 114 |
| 31 | limit 30 to humans | 73 |
| 32 | limit 31 to english language | 72 |

Database(s): **Embase**1974 to 2023 Week 45
Search Strategy:

| **#** | **Searches** | **Results** |
| --- | --- | --- |
| 1 | exp Deep Learning/ | 46759 |
| 2 | "Deep Learning".tw. | 54870 |
| 3 | exp Artificial Intelligence/ | 89179 |
| 4 | "Artificial Intelligence".tw. | 41573 |
| 5 | exp Machine Learning/ | 427891 |
| 6 | "Machine Learning".tw. | 102317 |
| 7 | "Convolutional Neural Network*".tw. | 27987 |
| 8 | "Recurrent Neural Network*".tw. | 4711 |
| 9 | "Deep Neural Network*".tw. | 10338 |
| 10 | "Artificial Neural Network*".tw. | 20163 |
| 11 | "Deep Belief Network*".tw. | 474 |
| 12 | "Autoencoder*".tw. | 3376 |
| 13 | "Generative Adversarial Network*".tw. | 3477 |
| 14 | "Long Short-Term Memory Network*".tw. | 665 |
| 15 | "Computer Vision".tw. | 8032 |
| 16 | VGGNet.tw. | 173 |
| 17 | ResNet.tw. | 3011 |
| 18 | exp Time-Lapse Imaging/ | 15738 |
| 19 | "Time-Lapse*".mp. [mp=title, abstract, heading word, drug trade name, original title, device manufacturer, drug manufacturer, device trade name, keyword heading word, floating subheading word, candidate term word] | 23121 |
| 20 | exp Fertilization in Vitro/ | 94706 |
| 21 | "Fertilization in Vitro".tw. | 793 |
| 22 | "IVF".tw. | 52775 |
| 23 | "ICSI".tw. | 21420 |
| 24 | "Assisted Reproductive Techn*".tw. | 18573 |
| 25 | "Embryo*".tw. | 469837 |
| 26 | "Blastocyst*".tw. | 39763 |
| 27 | 1 or 2 or 3 or 4 or 5 or 6 or 7 or 8 or 9 or 10 or 11 or 12 or 13 or 14 or 15 or 16 or 17 | 493330 |
| 28 | 20 or 21 or 22 or 23 or 24 or 25 or 26 | 534925 |
| 29 | 18 or 19 | 32542 |
| 30 | 27 and 28 and 29 | 416 |
| 31 | limit 30 to humans | 371 |
| 32 | limit 31 to english language | 369 |

| **Database** | **Query** | **Results** |
| --- | --- | --- |
| **Scopus** | ( TITLE-ABS-KEY ( ( "Deep Learning" OR "Artificial Intelligence" OR "Machine Learning" OR "Convolutional Neural Network" OR "Convolutional Neural Networks" OR "Recurrent Neural Network" OR "Recurrent Neural Networks" OR "Deep Neural Network" OR "Deep Neural Networks" OR "Artificial Neural Network" OR "Artificial Neural Networks" OR "Deep Belief Network" OR "Deep Belief Networks" OR "Autoencoder*" OR "Generative Adversarial Network*" OR "Long Short-Term Memory Network*" OR "Computer Vision" OR vggnet OR resnet ) ) AND ALL ( ( "Time-Lapse*" OR "Time Lapse*" ) ) AND TITLE-ABS-KEY ( ( "Fertilization in Vitro" OR "in Vitro Fertilization" OR "IVF" OR "ICSI" OR "Intracytoplasmic Sperm Injection" OR "Assisted Reproductive Techn*" OR "Embryo*" OR "Blastocyst*" ) ) ) AND ( LIMIT-TO ( LANGUAGE , "English" ) ) AND ( EXCLUDE ( DOCTYPE , "ed" ) OR EXCLUDE ( DOCTYPE , "le" ) OR EXCLUDE ( DOCTYPE , "no" ) ) AND ( EXCLUDE ( EXACTKEYWORD , "Nonhuman" ) OR EXCLUDE ( EXACTKEYWORD , "Animals" ) OR EXCLUDE ( EXACTKEYWORD , "Animal" ) OR EXCLUDE ( EXACTKEYWORD , "Mouse" ) OR EXCLUDE ( EXACTKEYWORD , "Mice" ) OR EXCLUDE ( EXACTKEYWORD , "Animal Experiment" ) ) | 286 |
| **IEEE Xplore** | ("Deep Learning" OR "Artificial Intelligence" OR "Machine Learning" OR "Convolutional Neural Network" OR "Convolutional Neural Networks" OR "Recurrent Neural Network" OR "Recurrent Neural Networks" OR "Deep Neural Network" OR "Deep Neural Networks" OR "Artificial Neural Network" OR "Artificial Neural Networks" OR "Deep Belief Network" OR "Deep Belief Networks" OR "Autoencoder*" OR "Generative Adversarial Network*" OR "Long Short-Term Memory Network*" OR "Computer Vision" OR VGGNet OR ResNet) AND ("Time-Lapse*" OR "Time Lapse*") AND ("Fertilization in Vitro" OR "in Vitro Fertilization" OR "IVF" OR "ICSI" OR "Intracytoplasmic Sperm Injection" OR "Assisted Reproductive Techn*" OR "Embryo*" OR "Blastocyst*") | 26 |
| **ACM Digital library** | [[Full Text: "deep learning"] OR [Full Text: "artificial intelligence"] OR [Full Text: "machine learning"] OR [Full Text: "convolutional neural network"] OR [Full Text: "convolutional neural networks"] OR [Full Text: "recurrent neural network"] OR [Full Text: "recurrent neural networks"] OR [Full Text: "deep neural network"] OR [Full Text: "deep neural networks"] OR [Full Text: "artificial neural network"] OR [Full Text: "artificial neural networks"] OR [Full Text: "deep belief network"] OR [Full Text: "deep belief networks"] OR [Full Text: "autoencoder*"] OR [Full Text: "generative adversarial network*"] OR [Full Text: "long short-term memory network*"] OR [Full Text: "computer vision"] OR [Full Text: "vggnet" "resnet"]] AND [[Full Text: "fertilization in vitro"] OR [Full Text: "in vitro fertilization"] OR [Full Text: "ivf" "icsi"] OR [Full Text: "intracytoplasmic sperm injection"] OR [Full Text: "assisted reproductive techn*"] OR [Full Text: "embryo*" "blastocyst*"]] AND [[Full Text: "time-lapse*"] OR [Full Text: "time lapse*"]] | 19 |
| **Google Scholar** | ("Deep Learning" OR "Artificial Intelligence" OR "Machine Learning" OR "Convolutional Neural Network*" OR "Recurrent Neural Network*" OR "Deep Neural Network*" OR "Artificial Neural Network*") AND ("Time-Lapse*" OR "Time Lapse*") AND ("Fertilization in Vitro" OR "Fertilization in Vitro" OR "IVF" OR "ICSI" OR "Intracytoplasmic Sperm Injection" OR "Assisted Reproductive Techn*" OR "Embryo*" OR "Blastocyst*"). | 100 |
