# Supplementary material for: Deep learning applications for human embryo assessment using time-lapse imaging: scoping review
Source: Front Reprod Health. 2025 Apr 8;7:1549642. doi: 10.3389/frph.2025.1549642 (PMC12011738; doi:10.3389/frph.2025.1549642)
Supplement: Supplementary file 3 [file Table3.docx]

| **Extracted data** | **Definition** |
| --- | --- |
| **1. Study Characteristics** | |
| 1.1 Author | The first author of the study. |
| 1.2 Year of publication | The year in which the study was published. |
| 1.3 Type of publication | The venue where the study was published: peer-reviewed journal, book chapters, dissertations, or conference proceedings. |
| 1.4 Country of publication | The country where the study was published. |
| 1.5 Research design | The research design or methodology employed in a study, which can be categorized as:   - Retrospective - Prospective - Retrospective and Prospective |
| 1.6 Number of sites | The number of clinics involved in the study.   - 1 - 2 - 3 - 4 - >4 |
| 1.7 Number of participants | The number of women undergoing IVF procedure, or number of IVF cycles included. |
| 1.8 Mean age of participants | The average age of the participants in the study. |
| 1.9 Age range of participants | The range of ages of the participants in the study, indicating the minimum and maximum ages. |
| 1.10 Data sources | - Public: publicly available datasets that anyone can access, use, and share, often free of charge. - Private: proprietary datasets with restricted access, often controlled by licensing agreements or other legal constraints. - Public and private |
| **2. Applications and Outcomes** | |
| 2.1 Main application | 1. Embryo Development, Quality Assessment, and Grading 2. Embryo Chromosomal Composition 3. Pregnancy and Implantation Prediction |
| 2.2 Specific application | 1. **Embryo Development, Quality Assessment, and Grading**    - **Morphologic and morphometric parameters assessment**: analyzing the morphological and morphometric characteristics of preimplantation embryos, from the pronuclear stage to the blastocyst stage focusing on cell division, symmetry, and other morphological features.    - **Morphokinetic parameters assessment:** analyzing morphokinetic parameters of embryo development from the pronuclear to the blastocyst stage. Morphokinetics refers to the timing and dynamics (sequence of developmental events) of cell division and development.    - **Blastocyst Grading**: focuses solely on the blastocyst stage. It includes assessing the: (1) **Inner Cell Mass (ICM) Grading**: assessing the quality of the inner cell mass, which will form the fetus. (2) **Trophectoderm (TE) Grading**: assessing the quality of the trophectoderm, which will form the placenta and other supporting fetal structures.    - **Usable Blastocysts**: Identifying blastocysts suitable for transfer or freezing. 2. **Embryo chromosomal composition:**    - **Ploidy status:** predicting the ploidy status of the embryo. This means determining whether the embryo has the correct number of chromosomes. 3. **Pregnancy and Implantation Prediction**  - **Clinical Pregnancy or FH Pregnancy Prediction**: predicting the probability of achieving a full heartbeat-confirmed pregnancy at or beyond 7 weeks gestation. - **Live-Birth Prediction:** predicting the probability of the embryo resulting in a live birth. - **Miscarriage Rate**: predicting the probability of a spontaneous loss of clinical pregnancy before 20 weeks' gestation. - **Implantation Rate**: predicting the rate at which embryos successfully implant in the uterus. |
| 2.3 Outcome measures | Outcome used to evaluate the performance and effectiveness of the algorithm, such as:   1. **Embryo/Blastocyst morphology quality/grading**: evaluation of the physical characteristics and developmental stages of embryos and blastocysts, such as: **early embryo stages** (pre-blastocyst) [cell number and symmetry, fragmentation level], **blastocyst stage** [ICM quality and TE quality], and **overall grade assignment**. 2. **Blastocyst development**: evaluating whether the embryo reaches the blastocyst stage, the rate of blastulation, and other developmental milestones like time to reach the blastocyst stage or cell division rates. 3. **Ploidy status**: This refers to the assessment of the chromosomal composition of the embryo, determining whether it has the normal number of chromosomes (euploidy) or abnormalities (aneuploidy). 4. **Successful IVF** (Implantation Rate, Birth Prediction, Fetal Heartbeat on Ultrasound, miscarriage rate). |
| 2.4 Reference standard | Reference used to create the target variable (ground truth), such as:   - Embryologists - Ultrasound (for implantation and FH pregnancy) - Live-birth delivery (for live-birth rate) - Physicians |
| **3. Embryology and Time-lapse Platforms Characteristics** | |
| 3.1 Number of embryos | Number of embryos used in the study. |
| - 1. Embryo population | Developmental stage of the included embryo population:   - Pronuclear Stage (D1): Embryos with two pronuclei visible, indicating successful fertilization, observed on Day 1. - Cleavage (D1-D4): Embryos undergoing cell division, increasing in cell number without forming a blastocyst, from Day 1 to Day 4. - Blastocyst (D5, D6, D7): Embryos reaching the blastocyst stage, characterized by a fluid-filled cavity and cell differentiation, on Days 5 to 7. |
| 3.3 Time-lapse technology used | The specific time-lapse imaging system or technology utilized to monitor and record embryo development in the study, such as: EmbryoScope™, Primo Vision™, Miri® TL, GERI™. |
| 3.4 Time-lapse interval | Intervals at which images are captured in time-lapse embryo monitoring.   - Every 5 minutes - Every 10 minutes - Every 15 minutes - Every 20 minutes - Every 30 minutes - Every 60 minutes |
| 3.5 Annotation standards | The methods or criteria used by embryologists to classify and describe the developmental stages, quality, and other characteristics of embryos, such as:   - **Gardner's Blastocyst Grading System**: Widely used system for grading blastocysts based on the degree of expansion, inner cell mass quality, and trophectoderm quality. - **ASEBIR** (Association for the Study of Reproductive Biology): Classification criteria based on morphological assessment of embryos. - **Alpha ESHRE Consensus**: Developed by the Special Interest Group of Embryology under ESHRE, providing standardized terminology for embryo development and quality. |
| 3.6 Commercial Software | Commercial software used for embryo grading and classification (if any). |
| 1. **AI Methods Characteristics** | |
| 4.1 Training data | Types of data used to train the algorithm:   - **Image features:** Includes time-lapse image sequences, videos, or any other features related to the images (e.g., tPNf: time of pronuclei fading, tM: morula time, tSB: sub-blastocyst formation time, tB: blastocyst formation time). - **Demographics**: Patient’s age, ethnicity, and BMI. - **Clinical and reproductive history**: Number of previous pregnancies, number of previous live births, number of previous IVF cycles, and reasons for infertility. - **IVF treatment parameters**: FSH dose, number of oocytes retrieved, fertilization rate, number of embryos obtained, number of blastocysts obtained, blastulation rate, and number of transferred blastocysts. - **Male data**: Male age, male BMI, and reasons for male infertility. |
| 4.2 Main deep learning architecture | The main deep learning architecture used, such as:   - CNN (Convolutional Neural Networks) - RNN (Recurrent Neural Networks) - Transformers |
| 4.3 Specific deep learning architecture | Details the particular models or variants of deep learning architecture employed, such as:   - **ResNet (Residual Networks):** A type of CNN known for its deep architecture and 'skip connections' that allow it to learn effectively even when the network is very deep. - **VGGNet:** A type of CNN known for its simplicity and depth. It's widely used in image recognition tasks. - **LSTM (Long Short Term Memory):** A type of RNN specifically designed to remember long-term dependencies and avoid the vanishing gradient problem. - **Others** |
| 5.3 Validation methods | The approach used to validate the DL algorithm (e.g., Hold-out cross-validation, K-fold cross-validation, Leave One Out cross-validation, external validation) |
| 5.4 Performance metrics | The measures used to evaluate the accuracy and effectiveness of the AI algorithm in the study, such as accuracy, sensitivity, specificity, positive predictive value, negative predictive value, AUC-ROC, or other performance measures of the DL algorithm. |
