# Supplementary material for: Deep learning applications for human embryo assessment using time-lapse imaging: scoping review
Source: Front Reprod Health. 2025 Apr 8;7:1549642. doi: 10.3389/frph.2025.1549642 (PMC12011738; doi:10.3389/frph.2025.1549642)
Supplement: Supplementary file 7 [file Table7.docx]

**Multimedia Appendix 7 Deep Learning Models Characteristics.**

| **Study**  **ID** | **Author** | **Input Training Data** | **Main deep learning architecture** | **Specific deep learning architecture** | **DL algorithm Type pf validation** | **Performance metrics** |
| --- | --- | --- | --- | --- | --- | --- |
| 1 | Abbasi [1] | Image features | CNN | Unspecified CNN | K-fold | ACC, PREC, REC, Jaccard-Index |
| 2 | Abbasi [2] | Image features | CNN | ResNet | K-fold | ACC, PREC, REC, Jaccard-Index |
| 3 | Ahlstrom [3] | Image features | CNN | I3D | hold-out | AUC-ROC |
| 4 | Bamford [4] | Image features, demographics, clinical and reproductive history, ART parameters , male data | DNN | MLP | K-fold | SENS, SPES,FPR, AUC-ROC, ACC,PREC,F1 |
| 5 | Benchaib [5] | Image features, demographics , ART parameters | DNN, RNN | MLP, LSTM | K-fold | AUC-ROC, ACC, PREC,REC,F1 |
| 6 | Berntsen [6] | Image features | CNN | I3D | K-fold | AUC-ROC |
| 7 | Bori [7] | Image features | DNN | MLP | hold-out | AUC-ROC |
| 8 | Bormann [8] | Image features | CNN | CNNg | hold-out | AUC-ROC |
| 9 | Boucret [9] | Image features | NA | NA | hold-out | AUC-ROC |
| 10 | Chavez-Badiola [10] | Image features | DNN | Unspecified DNN | K-fold | AUC-ROC |
| 11 | Chen [11] | Image features , demographics, Clinical and reproductive history | CNN | AMSNet, AMCFNet | hold-out | AUC-ROC |
| 12 | Cimadomo [12] | Image features | CNN | I3D | hold-out | AUC-ROC |
| 13 | Cimadomo [13] | Image features | NA | NA | K-fold | p-value |
| 14 | Cimadomo [14] | Image features | NA | NA | K-fold | p-value |
| 15 | Coticchio [15] | Image features | RNN | LSTM | K-fold | SENS, SPES, ACC,PREC,F1 |
| 16 | Danardono [16] | Image features | CNN | Inception, EfficientNet, MobileNet, ResNet | hold-out | ACC |
| 17 | Danardono [17] | Image features | CNN | VGG, DenseNet | hold-out | REC, ACC,PREC,F1 |
| 18 | Dehkordi [18] | Image features | CNN | ResNet, Unet | hold-out | ACC, PREC, REC |
| 19 | Diakiw [19] | Image features, demographics | CNN | R-CNN, ResNet | K-fold | ACC,AUC-ROC,SENS |
| 20 | Diakiw [20] | Image features | CNN | ResNet, DeneNet | hold-out | ROC,AUC-ROC,ACC |
| 21 | Dirvanauskas [21] | Image features | CNN | AlexNet | hold-out | ACC |
| 22 | Duval [22] | Image features, demographics, clinical and reproductive history , ART parameters , male data | CNN | ResNet | K-fold | AUC-ROC,ACC,ROC,SENS |
| 23 | Eastick [23] | Image features | DNN | Unspecified DNN | hold-out | p-value |
| 24 | Einy [24] | Image features | CNN, RNN | LBCNN, LSTM | hold-out | ACC, AUC-ROC,PREC, F1 |
| 25 | Ezoe [25] | Image features | CNN | I3D | hold-out | p-value |
| 26 | Ferrick [26] | Image features | CNN | I3D | K-fold | p-value |
| 27 | Fukunaga [27] | Image features | NA | NA | hold-out | ACC,SENS |
| 28 | Gomez [28] | Image features | CNN, RNN | LSTM, ResNet | K-fold | r, ACC |
| 29 | Hammer [29] | Image features | CNN | Xception, ResNet | K-fold | ACC |
| 30 | Hori [30] | Image features | DNN | Unspecified DNN | K-fold | NA |
| 31 | Huang [31] | Image features, demographics | CNN | ResNet | K-fold | AUC-ROC,ACC |
| 32 | Huang [32] | Image features | CNN | ResNet | hold-out, K-fold | AUC-ROC |
| 33 | Huang [33] | Image features | CNN | Unet | hold-out | p-value |
| 34 | Johansen [34] | Image features | CNN | I3D | hold-out | AUC-ROC |
| 35 | Kallipolitis [35] | Image features | CNN | EfficientNet, Unet, ResNet | K-fold | ACC,AUC-ROC |
| 36 | Kanakasabapathy [36] | Image features | CNN | Xception | hold-out | AUC-ROC,ACC |
| 37 | Kato [37] | Image features | CNN | I3D | hold-out | AUC-ROC |
| 38 | Khan [38] | Image features | CNN | Unspecified CNN | K-fold | ACC |
| 39 | Khosravi [39] | Image features | CNN | Inception | hold-out | ACC, AUC-ROC |
| 40 | Kragh [40] | Image features | CNN,RNN | Xception, LSTM | hold-out | ACC,MSE, AUC-ROC |
| 41 | Kragh [41] | Image features | CNN | I3D | K-fold | AUC-ROC |
| 42 | Lassen [42] | Image features, demographics | CNN | I3D | hold-out | AUC-ROC |
| 43 | Leahy [43] | Image features | CNN | R-CNN, ResNet | hold-out | ACC,PREC |
| 44 | Lee [44] | Image features | CNN | I3D | hold-out | AUC-ROC,PREC |
| 45 | Liao [45] | Image features | CNN, RNN | DenseNet, LSTM | hold-out | AUC-ROC, ACC |
| 46 | Liu [46] | Image features | CNN | ResNet | hold-out | AUC-ROC,ACC |
| 47 | Liu [47] | Image features | CNN | ResNet, AlexNet | hold-out | MSE, ACC |
| 48 | Lockhart [48] | Image features | CNN | Unet | K-fold | ACC |
| 49 | Lukyanenko [49] | Image features | CNN,RNN | ResNet, LSTM | hold-out | ACC |
| 50 | Mapstone [50] | Image features | CNN | MobileNet | hold-out | AUC-ROC, ACC |
| 51 | Marsh [51] | Image features | CNN | ResNet, DenseNet | hold-out | AUC-ROC |
| 52 | Milewski [52] | Image features, demographics | DNN | MLP | hold-out | AUC-ROC |
| 53 | Nagaya [53] | Image features | CNN | ResNet | k-fold | AUC-ROC,PREC,FPR,NPV |
| 54 | Nguyen [54] | Image features | CNN | ResNet | K-fold | AUC-ROC,PREC,ACC |
| 55 | Ou [55] | Image features | CNN, Transformer | AlexNet, EfficientNet, Swin-T | hold-out | ACC,SENS,PPV,NPV,SPES |
| 56 | Papamentzelopoulou [56] | Image features | CNN | I3D | hold-out | AUC-ROC, p-value |
| 57 | Patil [57] | Image features | CNN | Unspecified CNN | hold-out | SPES,SENS,ACC |
| 58 | Paya [58] | Image features | CNN | ResNet, VGG, MLP | hold-out | ACC,PREC,SENS,SPES,F1,AUC-ROC |
| 59 | Rajendran [59] | Image features, demographics | CNN, RNN | LSTM, VGG | K-fold | AUC-ROC,MAE,ACC |
| 60 | Raudonis [60] | Image features | CNN | AlexNet, VGG | K-fold | ACC,PREC |
| 61 | Rocha [61] | Image features | DNN | Unspecified DNN | hold-out | ACC |
| 62 | Sawada [62] | Image features | CNN | ABN | K-fold | AUC-ROC |
| 63 | Sharma [63] | Image features | CNN, Transformer | DETR,YOLO | hold-out | PREC,AUPRC, SPES,ACC,F1, MCC |
| 64 | Thirumalaraju [64] | Image features | CNN | Iception, ResNet , NASNet, Xception | hold-out | AUC-ROC, ACC,SENS,SPES |
| 65 | Tran [65] | Image features | DNN | Unspecified DNN | K-fold, hold-out | AUC-ROC |
| 66 | Tran [66] | Image features | CNN | YOLO | hold-out | ACC,PREC |
| 67 | Ueno [67] | Image features | CNN | I3D | hold-out | AUC-ROC |
| 68 | Uysal [68] | Image features | CNN | Unet | K-fold | Jaccard-index,Dice,ACC,PREC |
| 69 | Vaidya [69] | Image features | CNN,RNN | LSTM, VGG, Xception,Inception, MobileNet, DenseNet, GRU | hold-out | MSE,ACC |
| 70 | Vergos [70] | Image features | CNN | AlexNet ,VGG | hold-out | PREC,F1,ACC |
| 71 | Wang [71] | Image features | CNN | DeepLab | hold-out | p-value |
| 72 | Wang [72] | Image features | CNN, Transformer | BYOL, IVFormer | k-fold | AUC-ROC, SPES |
| 73 | Xie [73] | Image features | CNN | AMSNet, TSM,MFS,ResNet | hold-out | AUC-ROC, ACC |
| 74 | Yuan [74] | Image features, demographics, ART parameters | CNN | Unspecified CNN | hold-out | AUC-ROC,SEN, SPEC, p-value |
| 75 | Zhao [75] | Image features | CNN | Unspecified CNN | K-fold | PREC,ACC |
| 76 | Zhu [76] | Image features, Clinical and reproductive history | CNN | I3D | hold-out | AUC-ROC |
| 77 | Zou [77] | Image features, demographics, ART parameters, male data | RNN | LSTM | K-fold | AUC-ROC,ACC,F1,PREC, AUPRC |

**References:**

1. Abbasi, M., et al.,, *A Deep Learning Approach for Prediction of IV Implantation Outcome from Day 3 and Day 5 Time-Lapse Human Embryo Image Sequences*, in *2021 IEEE International Conference on Image Processing (ICIP)*. 2021. p. 289-293.

2. Abbasi, M., et al., *Timed Data Incrementation: A Data Regularization Method for IVF Implantation Outcome Prediction from Length Variant Time-lapse Image Sequences*, in *2021 IEEE 23rd International Workshop on Multimedia Signal Processing (MMSP)*. 2021. p. 1-5.

3. Ahlstrom, A., et al., *Correlations between a deep learning-basedalgorithm for embryo evaluation with cleavage-stage cell numbers and fragmentation.* Reproductive BioMedicine Online, 2023. **47**(6).

4. Bamford, T., et al., *A comparison of 12 machine learning models developed to predict ploidy, using a morphokinetic meta-dataset of 8147 embryos.* Hum Reprod, 2023. **38**(4): p. 569-581.

5. Benchaib, M., et al., *Shallow artificial networks with morphokinetic time-lapse parameters coupled to ART data allow to predict live birth.* Reprod Med Biol, 2022. **21**(1): p. e12486.

6. Berntsen, J., et al., *Robust and generalizable embryo selection based on artificial intelligence and time-lapse image sequences.* PLoS One, 2022. **17**(2): p. e0262661.

7. Bori, L., et al., *An artificial intelligence model based on the proteomic profile of euploid embryos and blastocyst morphology: a preliminary study.* Reprod Biomed Online, 2021. **42**(2): p. 340-350.

8. Bormann, C.L., et al., *Performance of a deep learning based neural network in the selection of human blastocysts for implantation.* Elife, 2020. **9**.

9. Boucret, L., et al., *Change in the Strategy of Embryo Selection with Time-Lapse System Implementation-Impact on Clinical Pregnancy Rates.* J Clin Med, 2021. **10**(18).

10. Chavez-Badiola, A., et al., *Deep learning as a predictive tool for fetal heart pregnancy following time-lapse incubation and blastocyst transfer.* Hum Reprod, 2020. **35**(2): p. 482.

11. Chen, F., et al., *Knowledge-embedded spatio-temporal analysis for euploidy embryos identification in couples with chromosomal rearrangements.* Chin Med J (Engl), 2024. **137**(6): p. 694-703.

12. Cimadomo, D., et al., *Towards Automation in IVF: Pre-Clinical Validation of a Deep Learning-Based Embryo Grading System during PGT-A Cycles.* J Clin Med, 2023. **12**(5).

13. Cimadomo, D., et al., *How slow is too slow? A comprehensive portrait of Day 7 blastocysts and their clinical value standardized through artificial intelligence.* Hum Reprod, 2022. **37**(6): p. 1134-1147.

14. Cimadomo, D., et al., *Human blastocyst spontaneous collapse is associated with worse morphological quality and higher degeneration and aneuploidy rates: a comprehensive analysis standardized through artificial intelligence.* Hum Reprod, 2022. **37**(10): p. 2291-2306.

15. Coticchio, G., et al., *Cytoplasmic movements of the early human embryo: imaging and artificial intelligence to predict blastocyst development.* Reprod Biomed Online, 2021. **42**(3): p. 521-528.

16. Danardono, G.B., et al., *A Homogeneous Ensemble of Robust Pre-defined Neural Network Enables Automated Annotation of Human Embryo Morphokinetics.* J Reprod Infertil, 2022. **23**(4): p. 250-256.

17. Danardono, G.B., et al., *Embryo ploidy status classification through computer-assisted morphology assessment.* AJOG Glob Rep, 2023. **3**(3): p. 100209.

18. Dehkordi, S., and Moghaddam, M.,, *The Detection of Blastocyst Embryo In Vitro Fertilization (IVF)*, in *International Conference on Machine Vision and Image Processing (MVIP),*. 2022: Ahvaz, Iran, Islamic Republic. p. 1-6.

19. Diakiw, S.M., et al., *Development of an artificial intelligence model for predicting the likelihood of human embryo euploidy based on blastocyst images from multiple imaging systems during IVF.* Hum Reprod, 2022. **37**(8): p. 1746-1759.

20. Diakiw, S.M., et al., *An artificial intelligence model correlated with morphological and genetic features of blastocyst quality improves ranking of viable embryos.* Reprod Biomed Online, 2022. **45**(6): p. 1105-1117.

21. Dirvanauskas, D., et al., *Embryo development stage prediction algorithm for automated time lapse incubators.* Comput Methods Programs Biomed, 2019. **177**: p. 161-174.

22. Duval, A., et al., *A hybrid artificial intelligence model leverages multi-centric clinical data to improve fetal heart rate pregnancy prediction across time-lapse systems.* Hum Reprod, 2023. **38**(4): p. 596-608.

23. Eastick, J., et al., *The presence of cytoplasmic strings in human blastocysts is associated with the probability of clinical pregnancy with fetal heart.* J Assist Reprod Genet, 2019. **38**(8): p. 2139-2149.

24. Einy, S., et al., *Local binary convolutional neural networks’ long short-term memory model for human embryos’ anomaly detection.* Sci Program, 2023.

25. Ezoe, K., et al., *Association between a deep learning-based scoring system with morphokinetics and morphological alterations in human embryos.* Reprod Biomed Online, 2022. **45**(6): p. 1124-1132.

26. Ferrick, L., Y.S.L. Lee, and D.K. Gardner, *Metabolic activity of human blastocysts correlates with their morphokinetics, morphological grade, KIDScore and artificial intelligence ranking.* Hum Reprod, 2019. **35**(9): p. 2004-2016.

27. Fukunaga, N., et al., *Development of an automated two pronuclei detection system on time-lapse embryo images using deep learning techniques.* Reprod Med Biol, 2020. **19**(3): p. 286-294.

28. Gomez, T., *Towards deep learning-powered IVF: A large public benchmark for morphokinetic parameter prediction.* ArXiv, 2022. **abs/2203.00531**.

29. Hammer, K.C., et al., *Using artificial intelligence to avoid human error in identifying embryos: a retrospective cohort study.* J Assist Reprod Genet, 2022. **39**(10): p. 2343-2348.

30. Hori, K., et al., *Comparison of euploid blastocyst expansion with subgroups of single chromosome, multiple chromosome, and segmental aneuploids using an AI platform from donor egg embryos.* J Assist Reprod Genet, 2023. **40**(6): p. 1407-1416.

31. Huang, B., et al., *An artificial intelligence model (euploid prediction algorithm) can predict embryo ploidy status based on time-lapse data.* Reprod Biol Endocrinol, 2021. **19**(1): p. 185.

32. Huang, B., et al., *Using deep learning to predict the outcome of live birth from more than 10,000 embryo data.* BMC Pregnancy Childbirth, 2022. **22**(1): p. 36.

33. Huang, T.T.F., et al., *Deep learning neural network analysis of human blastocyst expansion from time-lapse image files.* Reprod Biomed Online, 2021. **42**(6): p. 1075-1085.

34. Johansen, M.N., et al., *Comparing performance between clinics of an embryo evaluation algorithm based on time-lapse images and machine learning.* J Assist Reprod Genet, 2023. **40**(9): p. 2129-2137.

35. Kallipolitis, A., Tziomaka, M., Papadopoulos, D., and Maglogiannis, I.,, *Explainable computer vision analysis for embryo selection on blastocyst images*, in *IEEE-EMBS International Conference on Biomedical and Health Informatics (BHI)*. 2022: Ioannina, Greece. p. 1-4.

36. Kanakasabapathy, M.K., et al., *Development and evaluation of inexpensive automated deep learning-based imaging systems for embryology.* Lab Chip, 2019. **19**(24): p. 4139-4145.

37. Kato, K., et al.,, *Does embryo categorization by existing artificial intelligence, morphokinetic or morphological embryo selection models correlate with blastocyst euploidy rates?* Reproductive BioMedicine Online, 2023. **46**(2): p. 274-281.

38. Khan, A., S. Gould, and M. Salzmann. *Deep Convolutional Neural Networks for Human Embryonic Cell Counting*. in *Computer Vision – ECCV 2016 Workshops*. 2016. Cham: Springer International Publishing.

39. Khosravi, P., et al., *Deep learning enables robust assessment and selection of human blastocysts after in vitro fertilization.* NPJ Digit Med, 2019. **2**: p. 21.

40. Kragh, M.F., et al., *Automatic grading of human blastocysts from time-lapse imaging.* Comput Biol Med, 2019. **115**: p. 103494.

41. Kragh, M.F., et al., *Predicting Embryo Viability Based on Self-Supervised Alignment of Time-Lapse Videos.* IEEE Trans Med Imaging, 2022. **41**(2): p. 465-475.

42. Lassen, J.T., et al., *Development and validation of deep learning based embryo selection across multiple days of transfer.* Sci Rep, 2023. **13**(1): p. 4235.

43. Leahy, B.D., et al., *Automated Measurements of Key Morphological Features of Human Embryos for IVF*, in *Med Image Comput Comput Assist Interv*. 2020. p. 25-35.

44. Lee, C.I., et al., *End-to-end deep learning for recognition of ploidy status using time-lapse videos.* J Assist Reprod Genet, 2021. **38**(7): p. 1655-1663.

45. Liao, Q., et al., *Development of deep learning algorithms for predicting blastocyst formation and quality by time-lapse monitoring.* Commun Biol, 2021. **4**(1): p. 415.

46. Liu, H., et al.,, *Automated Morphological Grading of Human Blastocysts From Multi-Focus Images.* IEEE Transactions on Automation Science and Engineering, 2023.

47. Liu, Z., et al.,, *Multi-Task Deep Learning With Dynamic Programming for Embryo Early Development Stage Classification From Time-Lapse Videos.* IEEE Access, 2019. **7**: p. 122153-122163.

48. Lockhart, L., et al.,, *Human Embryo Cell Centroid Localization and Counting in Time-Lapse Sequences*, in *25th International Conference on Pattern Recognition (ICPR)*. 2020: Milan, Italy. p. 8306-8311.

49. Lukyanenko, S., et al., *Developmental Stage Classification of Embryos Using Two-Stream Neural Network with Linear-Chain Conditional Random Field.* Med Image Comput Comput Assist Interv, 2021. **12908**: p. 363-372.

50. Mapstone, C., et al.,, *Deep learning pipeline reveals key moments in human embryonic development predictive of live birth in IVF.* bioRxiv, 2023.

51. Marsh, P., et al., *A proof of concept for a deep learning system that can aid embryologists in predicting blastocyst survival after thaw.* Sci Rep, 2022. **12**(1): p. 21119.

52. Milewski, R., et al., *How much information about embryo implantation potential is included in morphokinetic data? A prediction model based on artificial neural networks and principal component analysis.* Adv Med Sci, 2017. **62**(1): p. 202-206.

53. Nagaya, M., and Ukita, n.,, *Embryo Grading With Unreliable Labels Due to Chromosome Abnormalities by Regularized PU Learning With Ranking.* IEEE Transactions on Medical Imaging, 2022. **41**.

54. Nguyen, T.P., et al.,, *EmbryosFormer: Deformable Transformer and Collaborative Encoding-Decoding for Embryos Stage Development Classification*, in *2023 IEEE/CVF Winter Conference on Applications of Computer Vision (WACV)*. 2023. p. 1980-1989.

55. Ou, Z., et al ., and *Classification of human embryos by using deep learning*, in *Other Conferences*. 2023.

56. Papamentzelopoulou, M.S., et al., *Assessment of artificial intelligence model and manual morphokinetic annotation system as embryo grading methods for successful live birth prediction: a retrospective monocentric study.* Reprod Biol Endocrinol, 2024. **22**(1): p. 27.

57. Patil, S.N., *Selection of Single Potential Embryo to Improve the Success Rate of Implantation in IVF Procedure using Machine Learning Techniques,*, in *International Conference on Communication and Signal Processing (ICCSP)*. 2019: Chennai, India. p. 0881-0886.

58. Paya, E., et al., *Automatic characterization of human embryos at day 4 post-insemination from time-lapse imaging using supervised contrastive learning and inductive transfer learning techniques.* Comput Methods Programs Biomed, 2022. **221**: p. 106895.

59. Rajendran, S., et al., *Automatic Ploidy Prediction and Quality Assessment of Human Blastocyst Using Time-Lapse Imaging.* bioRxiv, 2023.

60. Raudonis, V., et al., *Towards the automation of early-stage human embryo development detection.* Biomed Eng Online, 2019. **18**(1): p. 120.

61. Rocha, J.C., et al., *Using Artificial Intelligence to Improve the Evaluation of Human Blastocyst Morphology*, in *International Joint Conference on Computational Intelligence*. 2017.

62. Sawada, Y., et al., *Evaluation of artificial intelligence using time-lapse images of IVF embryos to predict live birth.* Reprod Biomed Online, 2021. **43**(5): p. 843-852.

63. Sharma, A.A., A.Z.; Kakulavarapu, R.; Stensen, M.H.; Riegler, M.A.; Hammer, H.L. , *Predicting Cell Cleavage Timings from Time-Lapse Videos of Human Embryos.* Big Data Cogn. Comput., 2023. **7**(91).

64. Thirumalaraju, P., et al., *Evaluation of deep convolutional neural networks in classifying human embryo images based on their morphological quality.* Heliyon, 2021. **7**(2): p. e06298.

65. Tran, D., et al., *Deep learning as a predictive tool for fetal heart pregnancy following time-lapse incubation and blastocyst transfer.* Hum Reprod, 2019. **34**(6): p. 1011-1018.

66. Tran, H.P., et al., *Microscopic Video-Based Grouped Embryo Segmentation: A Deep Learning Approach.* Cureus, 2023. **15**(9): p. e45429.

67. Ueno, S., et al., *Improved pregnancy prediction performance in an updated deep-learning embryo selection model: a retrospective independent validation study.* Reprod Biomed Online, 2023. **48**(1): p. 103308.

68. Uysal, N., et al.,, *Comparison of U-Net Based Models for Human Embryo Segmentation*, in *Bilişim Teknolojileri Dergisi*. 2022.

69. Vaidya, G., et al.,, *Time Series Prediction of Viable Embryo and Automatic Grading in IVF using Deep Learning.* The Open Biomedical Engineering Journal, 2021.

70. Vergos , G., et al.,, *Ensemble Learning Technique for Artificial Intelligence Assisted IVF Applications*, in *12th International Conference on Modern Circuits and Systems Technologies (MOCAST)*. 2023, IEEE: Athens, Greece.

71. Wang, S., L. Chen, and H. Sun, *Interpretable artificial intelligence-assisted embryo selection improved single-blastocyst transfer outcomes: a prospective cohort study.* Reprod Biomed Online, 2023. **47**(6): p. 103371.

72. Wang, G., et al., and *A generalized AI system for human embryo selection covering the entire IVF cycle via multi-modal contrastive learning.* Patterns, 2024.

73. Xie, X., et al.,, *Early Prediction of Blastocyst Development via Time-Lapse Video Analysis*, in *IEEE 19th International Symposium on Biomedical Imaging (ISBI)*. 2022: Kolkata, India. p. 1-5.

74. Yuan, Z., et al., *Development of an artificial intelligence based model for predicting the euploidy of blastocysts in PGT-A treatments.* Sci Rep, 2023. **13**(1): p. 2322.

75. Zhao, M., et al., *Application of convolutional neural network on early human embryo segmentation during in vitro fertilization.* J Cell Mol Med, 2021. **25**(5): p. 2633-2644.

76. Zhu, J., et al., *External validation of a model for selecting day 3 embryos for transfer based upon deep learning and time-lapse imaging.* Reprod Biomed Online, 2023. **47**(3): p. 103242.

77. Zou, Y., et al., *Can the combination of time-lapse parameters and clinical features predict embryonic ploidy status or implantation?* Reprod Biomed Online, 2022. **45**(4): p. 643-651.
